# Intranasally delivered fucoidan nanozyme–decorated photosynthetic *Chlamydomonas* regulates vascular pathology and hypoxia in diabetes-associated retinal microvascular dysfunction

**DOI:** 10.1016/j.mtbio.2026.103060

**Published:** 2026-03-26

**Authors:** Andrew E.-Y. Chuang, Yo-Lin Chen, Chia-Hung Liu, Hieu Trung Nguyen, Tsung-Jen Wang, I-Chan Lin

**Affiliations:** a Graduate Institute of Biomedical Materials and Tissue Engineering, International Ph.D. Program in Biomedical Engineering, College of Biomedical Engineering, Taipei Medical University, New Taipei City, 235603, Taiwan; b Cell Physiology and Molecular Image Research Center, Taipei Medical University, Wan Fang Hospital, Taipei, 11696, Taiwan; c Precision Medicine and Translational Cancer Research Center, Taipei Medical University Hospital, Taipei, 11031, Taiwan; d Department of Urology, School of Medicine, College of Medicine, Taipei Medical University, 250 Wu-Hsing Street, Taipei, 11031, Taiwan; e Taipei Medical University Research Center of Urology and Kidney, Taipei Medical University, 250 Wu-Hsing Street, Taipei, 11031, Taiwan; f Department of Urology, Shuang Ho Hospital, Taipei Medical University, 291 Zhongzheng Road, Zhonghe District, New Taipei City, 23561, Taiwan; g Department of Orthopedics and Trauma, Faculty of Medicine, University of Medicine and Pharmacy at Ho Chi Minh City, Ho Chi Minh City, 700000, Viet Nam; h Department of Ophthalmology, Taipei Medical University Hospital, Taipei, 11031, Taiwan; i Department of Ophthalmology, School of Medicine, College of Medicine, Taipei Medical University, Taipei, 11031, Taiwan; j Department of Ophthalmology, Wan Fang Hospital, Taipei Medical University, Taipei, 11031, Taiwan

**Keywords:** Diabetic retinopathy, Chlamydomonas@-Poly(3,4- ethylenedioxythiophene)/protamine/fucoidan nanozyme, Immunity, Mitigating hypoxia, Vascularization, P-selectin targeting

## Abstract

Diabetic retinopathy (DR) is a leading cause of vision loss and is strongly associated with microvascular pathology and retinal hypoxia. Here, we report an intranasally delivered microalgae drug delivery system, Chlamydomonas@Pro-Fu-PEDOT nanozymes (CHL@Pro-Fu-PEDOT-NZs), engineered to target retinal injury. Under 660-nm red-light irradiation, the platform exploits the photosynthetic activity of Chlamydomonas to generate bioelectric cues and therapeutic gases (O_2_), thereby alleviating hypoxia and reducing vascularization. Fucoidan functionalization further confers P-selectin–mediated vascular addressing, enabling lesion-focused delivery and regulation of hypoxia-driven vascular responses through photosynthesis-inspired gas generation and bioelectrical modulation. In vitro, CHL@Pro-Fu-PEDOT-NZs exhibited excellent biocompatibility and protected retinal ganglion cells from oxidative and inflammatory injury. In a streptozotocin (STZ)–induced DR mouse model, intranasal delivery mitigated pathological retinal responses, as evidenced by reduced VEGF and HIF-1α expression, and yielded measurable improvements in visual behavior, without detectable systemic toxicity. Collectively, this multifunctional, noninvasive therapeutic platform offers strong potential for DR treatment by simultaneously addressing vascular dysfunction, and hypoxia.

## Graphical abstract

**Figure:**
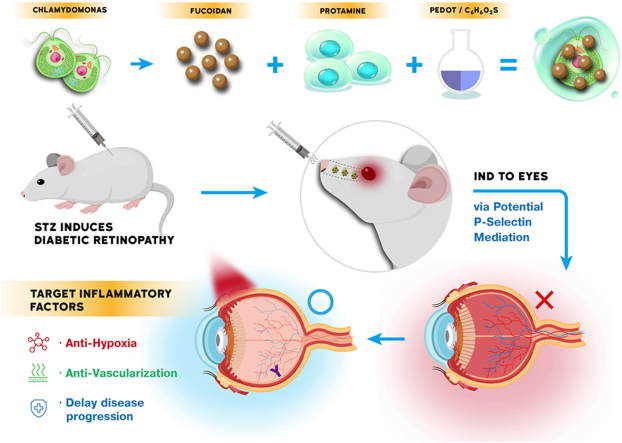

## 1 Introduction

Diabetic retinopathy (DR) is a major contributor to vision loss and stands as one of the most prevalent vascular impediments associated with diabetic disease in individuals of working age [1,2]. By 2045, the DR's global occurrence is expected to rise to 22% [3,4]. Research has extensively documented that a variety of mechanisms, including hypoxia, oxidative stress, inflammation, and dysfunction of retinal blood vessels drives the development of DR [[5], [6], [7], [8]].

In conditions of hyperglycemia, expression of hypoxia-inducible factor (HIF)-1α is upregulated, resulting in the transcriptional bioactivation of vascular endothelial growth factor (VEGF) [9], which stimulates vascularization within DR [10,11]. Elevated levels of the blood glucose limit oxygen (O_2_) supply to the intra-retinal vascular network, establishing a hypoxia microenvironment [12]. Hypoxia-driven pathways play a crucial role in retinal vascularization, as the activation of HIF-1α stimulates VEGF signaling, a key mediator responsible for abnormal blood vessel formation. [[13], [14], [15]]. Moreover, oxidative cellular stress and the buildup of the ROS due to hypoxia further aggravate endothelial dysfunction, promote aberrant vascularization [16,17], and amplify the intra-retinal inflammation [18,19]. The response of inflammation is characterized by increased extents of inflammatory cytokines, such as interleukin (IL-6), tumor necrosis factor (TNF-α), and IL-1β [20]. Clinical research has shown significantly higher levels of IL-1β, IL-6, TNF-α, and VEGF in the vitreous and plasma of DR patients, with these increases correlating with disease severity [21,22].

Current management for DR, such as anti-VEGF therapy, a vitrectomy, laser photocoagulation, and verteporfin-based photodynamic therapy, mainly focus on addressing the abnormal intra-retinal vascular network [[23], [24], [25]]. Although these treatments can slow progression of the disease, many patients continue to suffer from persistent fluid leakage, uncontrolled bleeding, and recurring hemorrhages [26]. Organic antioxidants, including betaine [27] and melatonin [16,28], have been investigated for their potential to alleviate oxidative stress-induced vascularization and reduce ROS. Despite this, their clinical use remains limited due to challenges such as low bioavailability, poor stability, and inadequate therapeutic effectiveness [29,30]. Therefore, there is a pressing need to develop innovative therapeutic approaches that meritoriously enhance the clearance of ROS and reduce oxidative damage to improve DR treatment outcomes.

The green alga *Chlamydomonas* (CHL) exhibits robust photosynthetic capabilities, allowing it to generate oxygen upon light exposure [31,32]. This dual functionality enables CHL to mitigate hypoxic conditions and inhibit pathological angiogenesis, making it a compelling candidate for regenerative medicine and targeted drug delivery applications [33]. Its multifunctional nature has garnered significant interest in the realm of precision biomedicine, particularly for enhancing therapeutic strategies in disorders associated with hypoxia [32,34]. Recent advancements in integrating of biocompatible conducting polymers into microalgal systems have demonstrably improved photosynthetic efficiency by optimizing light harvesting and electron/energy transfer processes [35]. Such enhancements not only boost overall photosynthetic performances but also expand the scope of potential applications in bioenergy, environmental remediation, and therapeutic interventions in biomedicine [36].

Intranasal administration (IND) offers an effective and minimally invasive method for delivering drugs to the eye, especially for treating of retinal diseases [37]. Recent research showed that IND facilitates drug delivery to the retina through the optic nerve pathway in glaucoma models, reducing related side effects and systemic exposure [38]. Eye medicines generally enter the ophthalmic tissues through two main pathways: the conjunctival route, linked to the blood-retinal barrier (including the sclera, conjunctiva, choroid, vitreous, and retina) [39], and the corneal route, which involves the blood-aqueous barrier (comprising the aqueous humor, intraocular tissues, and cornea) [40].

The previous work [41] employed a platelet cell–constructed microalgal system to demonstrate feasibility in an STZ-induced DR murine model, primarily focusing on the modulation of ocular inflammation and immune responses, with potential involvement of IL-6 as a biomarker. However, it did not comprehensively investigate behavioral outcomes, the photosynthetic oxygen-generating capacity for alleviating ocular hypoxia, or vascular-related biological interactions, all of which are critical for a thorough evaluation of DR progression and therapeutic efficacy. In addition, compared with polymeric biomaterials, platelet cell–based systems may face greater challenges in regulatory approval and clinical translation. Building upon these limitations, the present study should further evaluate live versus dead microalgal systems across different administration routes to systematically assess delivery efficiency and safety profiles. These considerations motivate the development of advanced polymeric biomaterial–based strategies to achieve a more comprehensive and translationally relevant intervention for DR.

Fucoidan-based nanozymes (Fu NZs) were shown to strongly bind to inflammatory retinal lesions, especially through interactions with P-selectin [42]. These vesicles effectively regulate immune and inflammatory responses and neutralize ROS, demonstrating their aptitude as the therapeutics approach for ophthalmology disorders associated with inflammation and oxidative cellular stress [[43], [44], [45]]. The integration of CHL with teal pigment–based conducting polymer–derived Fu NZs may offer a promising strategy for DR therapy, leveraging phototherapeutic oxygenation, enhanced photosynthetic efficiency, and targeted anti-inflammatory effects.

Recent studies indicate that while photothermal therapy (PTT) shows strong potential for tumor ablation, its standalone effectiveness remains limited. To enhance outcomes, nitric oxide (NO)-mediated gas therapy (GT) has emerged as a complementary approach due to NO's anti-inflammatory, vasodilatory, and cytotoxic effects. The integration of photothermal agents (PTAs) with thermally responsive NO donors enables synergistic PTT/GT under a single laser irradiation, improving therapeutic precision while minimizing gas toxicity. Additionally, advanced nanoplatforms capable of stimuli-responsive NO release—triggered by light, ultrasound, reactive oxygen species, or glutathione—have further enhanced treatment selectivity. Combining PTT/GT with chemotherapy, photodynamic therapy, radiotherapy, or immunotherapy has led to the development of multimodal nanotherapeutics with superior efficacy and reduced side effects [46].

**Scheme 1:**
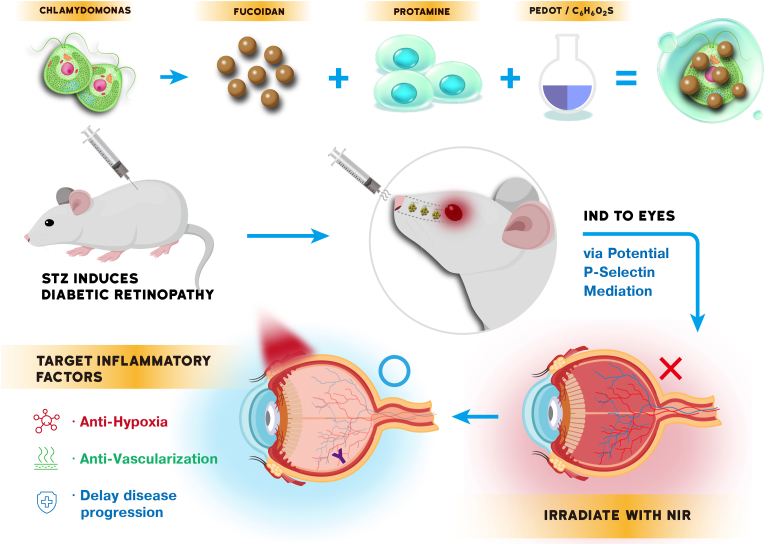
Schematic illustration of intranasal delivery (IND) of CHL@Pro-Fu-PEDOT-NZs for the treatment of diabetic retinopathy (DR). Chlamydomonas (CHL) was integrated with fucoidan nanozymes (Fu NZs), protamine (Pro), and PEDOT to form the CHL@Pro-Fu-PEDOT-NZ complex. DR was induced in a murine model using streptozotocin (STZ). The IND formulation was administered intranasally, enabling delivery to the retinal site. Following migration toward inflamed retinal lesions, near-infrared (NIR) irradiation activated photosynthetic processes, resulting in oxygen generation and bioelectric stimulation. This approach alleviated hypoxia, reduced oxidative stress, suppressed inflammatory responses, and ultimately promoted vascular normalization and preservation of retinal function.

In this study, we developed a biohybrid IND system (CHL@Pro-Fu-PEDOT-NZs) by incorporating Fu NZs into a biocompatible CHL-based platform, together with pigment-based PEDOT and protamine (Pro) as a stabilizing component. This integrated system was designed to address key pathological features of DR, including hypoxia and excessive reactive oxygen species (ROS) accumulation. Upon 660 nm light activation, CHL initiates photosynthetic processes, generating bioelectrons and oxygen to alleviate hypoxia and modulate inflammatory responses. PEDOT enhances electron transfer efficiency, while fucoidan facilitates targeting toward inflamed retinal tissues through potential P-selectin-mediated interactions, thereby improving retinal accumulation and penetration across physiological barriers. Collectively, this synergistic platform enables hypoxia alleviation, immune modulation, suppression of pathological vascular activity, and ROS scavenging, offering a promising and non-invasive phototherapeutic strategy for the treatment of DR (Scheme 1).

## 2 Experimental section

### 2.1 Materials

The materials used in this study included various chemicals and substances such as Prussian blue (PB), PEDOT, lipopolysaccharide (LPS), streptozotocin (STZ), Pro, a special dye called 4’,6-diamidino-2-phenylindole (DAPI), and other products like Fu and Amplex red, all sourced from Sigma-Aldrich (St. Louis, MO, USA). To prepare our solutions and samples, we used ultrapure water, which has very high purity (with a resistance exceeding 18.2 megohm cm^−1^), obtained from Millipore (Burlington, MA, USA). Unless noted otherwise, all experiments were conducted at room temperature, specifically 25 °C.

Human retinal ganglion cells (RGCs) and certain cell lines were sourced from American Type Culture Collection (Manassas, VA, USA), a well-known biological resource center. We used specific fluorescent antibodies, which helped identify certain proteins in our cells, to study different types of immune cells, particularly M1 and M2 macrophages. These antibodies, related to various proteins like VEGF and IL-6, were acquired from Solarbio Life Sciences (Beijing, China). Additionally, we utilized the luminescent oxygen sensor, (Ru(dpp)3]Cl2^47^), which we obtained from Asia Bioscience Co., Ltd. (Taiwan).

In our experiments, we used phosphate-buffered saline (PBS) to help maintain the right balance of salt and acidity, with very low salt levels and a specific amount of phosphate to maintain stability at pH 7.4, which is similar to the acidity found in the human body. Additionally, we obtained CHL from the Chlamydomonas Resource Center at the University of Minnesota (Minneapolis, MN, USA), which is known for its work with certain types of algae.

### 2.2 Formulation and physiochemical characterizations of CHL@Pro-Fu-PEDOT-NZs

#### 2.2.1 Optimization of Pro-Fu-PEDOT-NZs formulation

To refine the ratios of formulation for Pro-Fu-PEDOT-NZs, we systematically varied the PEDOT content while maintaining a constant mass ratio of Fu to Pro. Specifically, we prepared formulations with Fu:PEDOT:Pro mass ratios of 7.5:1:2.5, 7.5:2:2.5, 7.5:4:2.5, 7.5:6:2.5, and 7.5:8:2.5. Each formulation was mixed for 10 min at 300 rpm in a pH 7.4 aqueous solution under ambient conditions (1 atm, 22 °C) to ensure homogenous dispersion of all components. Following this, we conducted a dynamic light scattering (DLS) analysis using a DLS ZS90 (Malvern Panalytical, Malvern, UK) to assess the particle size distribution and zeta potential, facilitating a comprehensive evaluation of the optimal PEDOT/Pro/Fu composition.

#### 2.2.2 Integration of CHL with Pro-Fu-PEDOT-NZs nanoparticles

To optimize the formulations of integrated PEDOT/Pro/Fu and CHL, we adjusted the content of PEDOT while retaining a constant weighting ratio of Fu to Pro. Additionally, we maintained a 10^5^ cells/mL CHL of throughout the experiments. The resulting formulations exhibited CHL (10^5^ cells/mL): optimal PEDOT/Pro/Fu composition ratios of 1:1.2, 1:1.6, 1:2, 1:2.4, and 1:2.8. Each mixture was subjected to stirring at pH 7.4 and 300 rpm in an aqueous environment under ambient conditions (1 atm, 22 °C) to ensure optimal dispersion.

#### 2.2.3 Microscopy analysis of CHL cellular motility

Subsequently, we assessed the suspensions using a microscope (Leica) to investigate time-dependent variations in the live CHL cellular motility subjected to various weight ratio of optimized Pro-Fu-PEDOT-NZs.

#### 2.2.4 Particle size and zeta potential analysis

Additionally, we employed DLS (DLS ZS90, Malvern Panalytical) to evaluate changes in the zeta potential, providing insights into the colloidal stability of the different formulations in an aqueous environments. Our analysis encompassed free Pro-Fu-PEDOT-NZs, unmodified CHL cells, and the hybrid CHL@Pro-Fu-PEDOT-NZ.

#### 2.2.5 UV-Vis absorption spectroscopy

To analyze the absorbance characteristics of the different samples, we utilized an ultraviolet-visible spectrophotometer (SpectraMax 190, Molecular Devices, San Jose, CA, USA). to assess CHL, optimized Pro-Fu-PEDOT-NZs, and the composite CHL@Pro-Fu-PEDOT-NZs in the wavelength range of 500–800 nm.

#### 2.2.6 Electrochemical response evaluation via PB redox assay

To assess the electricity-generating capabilities of the materials under investigation—namely CHL, optimized Pro-Fu-PEDOT-NZs, and the composite CHL@Pro-Fu-PEDOT-NZs—each sample was combined with PB, an electrochromic agent, and analyzed using UV-Vis spectroscopy (SpectraMax 190 reader at 720 nm). The wavelength aligned with a specific absorption characteristic of PB, facilitating the observation of any alterations in its redox state upon interactions with the various samples, both in the presence and absence of 660-nm irradiation. By evaluating variations in intensity across individual components (CHL and optimized Pro-Fu-PEDOT-NZs) and their hybrid (CHL@Pro-Fu-PEDOT-NZs), we could derive important insights into the combinational effects and improved electricity originating from the complex.

#### 2.2.7 Oxygen generation assay using RDPP probe

Because RDPP is an oxygen-sensitive phosphorescent probe whose emission is dynamically quenched by dissolved O_2_, the fluorescence signal decreases as the local O_2_ concentration increases. Therefore, time-dependent changes in RDPP emission (Ex 450 nm/Em 630 nm) were used to monitor dissolved oxygen variations and to evaluate the net O_2_-generating (or O_2_-consuming) behavior of CHL, optimized Pro-Fu-PEDOT-NZs, and CHL@Pro-Fu-PEDOT-NZs under the specified conditions.

#### 2.2.8 Morphology and elemental composition analysis (SEM-EDS)

To investigate the morphological characteristics and elemental composition of the hybrid CHL@Pro-Fu-PEDOT-NZs, we employed scanning electron microscopy (SEM) in conjunction with energy-dispersive X-ray spectroscopy (EDS) (SEM-EDS; SU3500, Hitachi, Hitachinaka, Ibaraki, Japan). This synergistic methodology yielded high-resolution topographical images, elucidating surface morphologies and particle distributions. Additionally, elemental mapping facilitated the determination of spatial distributions and relative abundances of critical constituents within the structure.

#### 2.2.9 FTIR spectroscopy for chemical characterization

To conduct a comprehensive analysis of the chemical properties of individual components and the composite system, samples of CHL, Fu, Pro, PEDOT, and the hybrid CHL@Pro-Fu-PEDOT-NZs were analyzed using FTIR spectroscopy (Nicolet™ iS™ 10, Thermo Fisher Scientific, Waltham, MA, USA) provided vibrational signatures that indicated the presence of specific functional groups and potential bonding configurations.

### 2.3 In vitro tests

RAW 264.7 cells as well as RGCs were cultured in Dulbecco's modified Eagle medium (DMEM) supplemented with 100 μg/mL of streptomycin and 100 IU/mL of penicillin, along with 10% fetal bovine serum (Thermo Fisher Scientific). These cultures were maintained in a humidified incubator at 37 °C with 5% CO_2_ to promote optimal cellular growth and maintain viability.

#### 2.3.1 Cell viability (MTT assay) on RAW 264.7 cells and RGCs

For this experiment, we seeded 5000 cells/well in a 96-well plate. After a 0.5-day incubation period, cells were subjected to treatment with various formulations, some of which were paired with 660 nm illumination, based on the design of the specific study. Post-treatment, the medium was replaced with fresh cell medium supplemented with 3-(4,5-dimethylthiazol-2-Yl)-2,5-diphenyltetrazolium bromide (MTT, 0.5 mg/mL). Cells were subsequently incubated for a further 3 h, facilitating the conversion of MTT into purple formazan crystals within metabolically active cells. Following the removal of the cell medium, dimethyl sulfoxide (DMSO, 150 μL) was added to the cell well for solubilizing the formazan crystals. The plate was gently agitated for 10 min to achieve total dissolution, and the absorbance at 540 nm was then quantified on a microplate reader.

#### 2.3.2 Intracellular Uptake and chlorophyll fluorescence imaging in RGCs

To assess interactions of the CHL@Pro-Fu-PEDOT-NZ formulations with cells at inflammation situations, we monitored the red fluorescence emitted by chlorophyll as a key biomarker. RGCs were seeded in confocal dishes (6 × 10^4^ cells/dish). Following a 24-h adhesion period, we treated cells with the respective formulated groups in the absence or presence of lipopolysaccharide (LPS, Sigma Aldrich), which was utilized to induce an inflammatory state. Untreated RGCs served as a control group. Cells underwent an additional 24-h incubation period, after which they were counterstained with DAPI (Sigma Aldrich) to highlight nuclei. Fluorescence imaging was performed with a DMi8 microscope, allowing for real-time observation of DAPI-counterstained cellular nuclei and Chl-originated red fluorescence signals. This experimental arrangement enabled a detailed examination of interactions of the cell and biological responses amid inflammation stress.

#### 2.3.3 Inflammatory marker analysis in RGCs via IF and ROS detection

To further explore the effects of the designed groups on RGCs at inflammation stimulation, we subjected cells to treatments as previously detailed, incorporating either LPS stimulation or a control condition. Post-treatment, we assessed cells using a variety of inflammation markers, including immunofluorescence (IF) labeling for TNF-α and IL-6 and Amplex red for ROS detection. Cell nuclei were counterstained with DAPI to facilitate spatial visualization via fluorescence microscopy (DMi8). The fluorescence intensity of the respective signals was quantified with ImageJ software (National Institutes of Health, Bethesda, MD, USA), allowing for a detailed evaluation of how the different formulations influenced expressions of critical inflammation biomediators among RGCs.

#### 2.3.4 Macrophage polarization and inflammation marker analysis in RAW 264.7 cells

To assess the effects of various formulated groups on macrophage cells during inflammation responses, we subjected RAW 264.7 cells to treatment either with or without LPS, while adhering to established protocols. We then quantified key inflammatory markers: ROS were detected using Amplex red, while IF was employed to label M1 (CD86 or CD80) and M2 (CD206 or CD163) macrophage phenotypes, along with IL-6. Nuclei were stained by DAPI to ensure precise visualization. Fluorescence images were quantified with ImageJ, enabling precise comparison of anti-inflammatory and pro-inflammatory responses in RAW 264.7 macrophages under each treated situation.

### 2.4 In vivo tests

#### 2.4.1 Animal model establishment for diabetic retinopathy (DR)

Adult ICR mice were procured from BioLasco (Taipei, Taiwan). All procedures involving animal experimentation were subjected to review and approval by the Institutional Animal Care and Use Committee (IACUC) of Taipei Medical University (TMU) [SHLAC2023-0085, LAC2024-0395 and SHLAC2025-0087]. Experiments were conducted in accordance with TMU IACUC guidelines, ensuring the humane treatment of the animals through the administration of suitable anesthetics and analgesics.

To establish the diabetic retinopathy (DR) model, mice were administered streptozotocin (STZ) via intraperitoneal injection at a dose of 50 mg kg^−1^ day^−1^ for five consecutive days, which is a commonly used protocol for inducing diabetes in murine models [48]. Blood glucose levels were monitored regularly, and mice with glucose levels exceeding 200 mg dL^−1^ were considered diabetic and included in subsequent experiments.

For therapeutic treatment, mice received intranasal administration of a CHL@Pro-Fu-PEDOT-NZs suspension at an estimated dose of ∼2 mg kg^−1^ per administration, adjusted according to body weight. Following each administration, 660 nm laser irradiation (power density: 2.0 W cm^−2^, exposure time: 10 min) was applied, with the laser probe positioned approximately 10 cm from the nasal region to ensure uniform illumination and minimize the risk of localized overheating.

#### 2.4.2 Behavioral assessments: Morris water maze and gait analysis testing

To evaluate behavioral study after given CHL@Pro-Fu-PEDOT-NZs (IND) in mice with DR, we conducted a series of assessments including a Morris water maze test, and gait analysis. The Morris water maze test was performed using an apparatus provided by the Animal Center of Taipei Medical University, without a specific commercial model designation. (Taipei, Taiwan), for assessing spatial memory and learning, revealing cognitive functioning in the context of DR-induced stressors. The analysis of gait (ViewPoint Behavior Technology, Lyon, France) was utilized to examine locomotive posture and coordination, proposing crucial insights in neuromuscular bio-function. Collectively, these assessments facilitated a comprehensive evaluation of the neurological and visual benefits associated with CHL@Pro-Fu-PEDOT-NZs treatment in this DR mouse model.

#### 2.4.3 Biodistribution analysis via In vivo imaging (IVIS)

To investigate the biodistribution and metabolic dynamics of IND CHL@Pro-Fu-PEDOT-NZs in a DR mouse model, we employed an in vivo imaging system (IVIS (PerkinElmer IVIS Lumina III XRMS)) to monitor the spatial distribution of Chlorophyll-derivative red fluorescence across various tissues. For ex vivo imaging, the tumors and major organs were harvested and immediately subjected to fluorescence analysis using the IVIS Imaging System to evaluate tissue-specific fluorescence distribution. Specifically, in vivo fluorescence imaging was performed 24 h after intranasal administration of CHL@Pro-Fu-PEDOT-NZs in mice with STZ-induced diabetic retinopathy. By analyzing fluorescent signals associated with the Chlorophyll moiety, we assessed the clearance and localization kinetics of CHL@Pro-Fu-PEDOT-NZs in the context of DR pathology. This methodology afforded in-depth insights into the formulation's systemic distribution and metabolic fate over time.

#### 2.4.4 Histological and immunofluorescence analysis of systemic organs and eyes

To assess tissue-level responses following the IND of CHL@Pro-Fu-PEDOT-NZs in this DR mouse model, subjects were euthanized using CO_2_, and their heart, liver, spleen, lungs, kidneys, and eyes were excised for histological examinations. Hematoxylin and eosin (H&E) staining was performed on these tissues to identify any morphology changes or evidence of organ damage. For eye samples, supplementary IF analyses were conducted to evaluate expressions of specific biomarkers such as M1 (CD86) macrophages, IL-6 and M2 (CD206) macrophages, Amplex red, TNF-α, HIF-1α, P-selectin, and VEGF. Cellular nuclei were stained with DAPI to optimize visualization via microscopy. Fluorescence signals were quantified exploiting ImageJ software, giving a comprehensive analysis of the vascularization, inflammatory status, and structural integrity of the eyes and systemic organs.

##### 2.4.4.1 Hematological analysis and flow cytometry

To evaluate systemic safety, whole blood was collected from mice at the end of the treatment period into EDTA-coated tubes under anesthesia. Blood samples were diluted with PBS, and red blood cells were lysed when necessary using RBC lysis buffer. The resulting cell suspensions were analyzed using a flow cytometry-based hematology analyzer to assess major blood cell populations, including red blood cells (RBCs), platelets (PLTs), reticulocytes (RETs), and white blood cells (WBCs). Flow cytometry scatter profiles were generated based on forward scatter (FSC) and side scatter (SSC) parameters to evaluate cell size and granularity. All samples were analyzed under identical conditions to compare hematological distributions across experimental groups and assess potential systemic toxicity.

### 2.5 Statistical analysis

Comprehensive statistical analyses were performed using GraphPad Prism (La Jolla, CA, USA). Data are presented as mean ± standard deviation (SD), with each experimental group including a minimum of three independent replicates. Statistical significance was primarily evaluated using one-way ANOVA. When variance equality was not met, appropriate variance-corrected ANOVA methods were applied. Significance levels were defined as p < 0.05 (∗), p < 0.01 (∗∗), p < 0.001 (∗∗∗), and p < 0.0001 (∗∗∗∗). Sample sizes were determined based on preliminary data and prior studies employing similar nanotherapeutic strategies in diabetic retinopathy models, ensuring reliable statistical comparisons while adhering to the 3R (Replacement, Reduction, Refinement) principles for animal experimentation.

## 3 Results and discussion

### 3.1 Formulation and characterization physiochemical of CHL@Pro-Fu-PEDOT-NZs

#### 3.1.1 Physicochemical optimization of Pro-Fu-PEDOT-NZs and CHL complexes

**Fig. 1:**
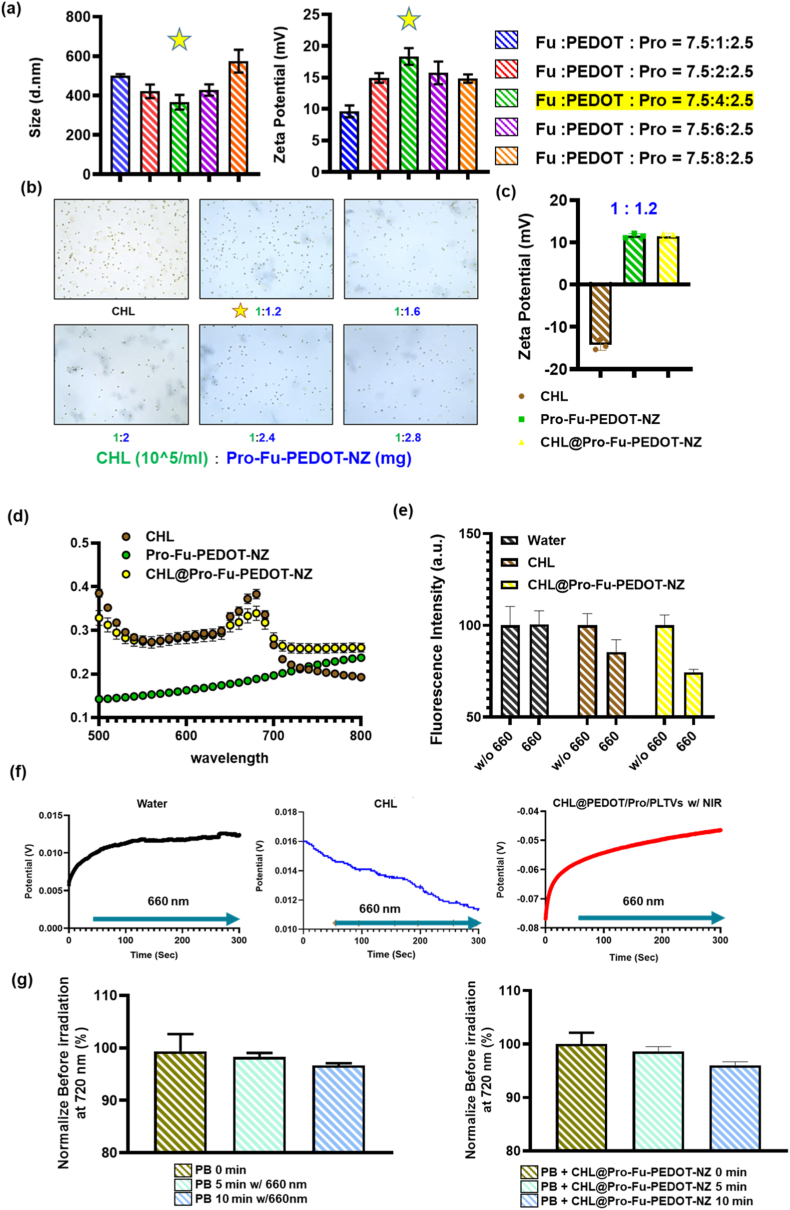
**Physicochemical optimization and photobioactive performance of Pro–Fu–PEDOT nanocomposites and CHL hybrids.** (a) Dynamic light scattering (DLS) analysis showing hydrodynamic diameter and zeta potential of Pro–Fu–PEDOT nanocomposites prepared at different mass ratios, identifying an optimized formulation (7.5:4:2.5; Pro–Fu–PEDOT-NZs). (b) Optical microscopy images (with **Supplementary Video**) assessing CHL motility after incubation with Pro–Fu–PEDOT-NZs at the indicated amounts, showing preserved swimming behavior at low loading and reduced motility at higher loading. (c) Zeta potential shift of CHL before and after complexation with Pro–Fu–PEDOT-NZs (CHL@Pro–Fu–PEDOT-NZ), indicating surface charge shift upon hybrid formation. (d) UV–Vis–NIR absorption spectra of CHL, Pro–Fu–PEDOT-NZs, and CHL@Pro–Fu–PEDOT-NZ. (e) RDPP-based dissolved oxygen sensing showing oxygen-dependent emission quenching and enhanced O_2_ evolution of CHL@Pro–Fu–PEDOT-NZs relative to free CHL under 660-nm illumination. (f) Photoelectrochemical output (bioelectricity) generated by CHL@Pro–Fu–PEDOT-NZs during 660 nm irradiation. (g) Prussian blue (PB) reduction assay monitored at 720 nm, indicating time-dependent electron accumulation/transfer during illumination of CHL@Pro–Fu–PEDOT-NZs. Data are presented as mean ± SD. (For interpretation of the references to colour in this figure legend, the reader is referred to the Web version of this article.)

DLS data shown in Fig. 1a demonstrate a decrease in particle size from approximately 500 nm to around 360 nm at mass ratios of 7.5:1:2.5, 7.5:2:2.5, and 7.5:4:2.5. Conversely, an increase in the average particle size to about 570 nm was observed at the higher loading ratio of 7.5:8:2.5 (Fig. 1a), indicating optimized aggregation and effective complexation among PEDOT, Pro, and Fu.

Additionally, the zeta potential displayed a transition from mildly positive values (+10 mV) at lower mass loadings to enhanced positive values at moderate loadings (7.5:1:2.5, 7.5:2:2.5, and 7.5:4:2.5), which correlated with the varying contributions of PEDOT in the resulting complexes. However, the zeta potential exhibited a slight reduction from approximately +16 to +14 mV at the higher mass ratios of 7.5:6:2.5 and 7.5:8:2.5. Given its smaller particle size, the 7.5:1:2.5 ratio was selected as optimized formulation for subsequent applications (Pro-Fu-PEDOT-NZs).

#### 3.1.2 Microscopy analysis of CHL motility with nanocomposites and surface charge interactions and enhanced absorption

The optical microscopic analysis presented in Fig. 1b, along with the **Supplementary Video**, indicates robust motility for free CHL and CHL combined with 1.2 mg of Pro-Fu-PEDOT-NZs. However, at elevated nanoparticle concentrations, a notable decrease in mobility was observed, likely due to particle aggregation that interfered with swimming dynamics. Fig. 1c illustrates changes in the zeta potential following interaction between CHL and Pro-Fu-PEDOT-NZs. The zeta potential shifted from an initial value of approximately −14 mV (CHL) to a positive value of +11 mV (CHL@Pro-Fu-PEDOT-NZ), likely attributed to the partial neutralization effects of the positively charged domains present in Pro. Furthermore, Fig. 1d demonstrates that the CHL@Pro-Fu-PEDOT-NZ composite preserved 660 nm absorption.

#### 3.1.3 Photosynthetic and photoelectrochemical performance assessment

The following assessments focused on photosynthesis dynamics and electron transfer efficiencies upon 660 nm treatment. Fig. 1e presents the assay of the RDPP fluorescence quenching, which reveals a significant quenching effect, indicating enhanced O_2_ evolution with CHL@Pro-Fu-PEDOT-NZs compared to free CHL. This increase in the photosynthetic performance suggests that the PEDOT-based complex promoted more-effective electron transfer, functioning similarly to a solar cell interface that enhanced CHL's intrinsic photosynthetic pathways. Additionally, electrical measurements corroborated the bioelectricity generated by CHL@Pro-Fu-PEDOT-NZs under 660 nm irradiation, as shown in Fig. 1f. Fig. 1g illustrates the photochromic alterations of PB at 720 nm, demonstrating a time-dependent accumulation of electrons during illumination of CHL@Pro-Fu-PEDOT-NZs, which implies sustained electron flow and intensified photochemical reactions.

#### 3.1.4 Synergistic photo-functionality

This mimetic structure not only promoted electron transfers but also advanced photosynthetic processes as well as enhanced the photochemical efficiency of conversion, opening pathways for uses in photobioreactors, bioenergy, and cutting-edge biomedicines. The oxygen-generating capacity of CHL under 660 nm irradiation was evaluated. As shown in the measurements, the dissolved oxygen level increased from 9.8 unit before irradiation to 17.8 unit after 660 nm exposure. This marked rise demonstrates that CHL can effectively enhance oxygen production upon light stimulation, highlighting its potential as a biological oxygen generator for therapeutic applications (Fig. S1).

#### 3.1.5 Mechanistic insights into component integration and functional effects

The incorporation of the therapeutic ingredients Fu, PEDOT, and CHL was facilitated by a biological molecular bridge formed by Pro (Protamine), a FDA-approved material [49,50]. This strategy ensured the stable assembly of these components into a cohesive hybrid, preserving colloidal stability while enhancing interfacial interactions among them. In this configuration, PEDOT functions analogously to a solar cell [51], significantly improving CHL's light-harvesting efficiency [52,53]. The enhanced photon absorption drives the photosynthetic process more effectively, resulting in elevated production of oxygen gases through the Calvin cycle. These gases [54,55] are recognized for displaying beneficial biochemical properties, including potent antioxidant and anti-inflammatory effects, as well as the ability to alleviate hypoxic microenvironments. Moreover, the effective transferring of electrons derived from photosynthesis to cell routes indicated that these biologically electric phenomena could enhance bioenergetics at the cellular level, facilitating procedures such as polarization of immune cells and regulation of comprehensive inflammation [56,57].

#### 3.1.6 Biomedical relevance and therapeutic potential

Recent studies highlighted that Fu NZs possess an inherent homing capability towards inflamed tissues [58], which positions them as a promising drug delivery vehicle. This characteristic enhances the efficiency of drug administration while potentially minimizing adverse effects and mitigating the necessity for frequent dosing schedules [59]. The synergistic incorporation of these elements within the CHL@Pro-Fu-PEDOT-NZ system signifies a forward-thinking strategy in the development of advanced photoredox platforms. These platforms not only show potential in applications such as regenerative medicine and bioenergy but also provide an innovative therapeutic framework for managing inflammation-related illnesses through targeted and meticulous interventions.

##### 3.1.6.1 Morphological and elemental characterization of CHL@Pro-Fu-PEDOT-NZs

**Fig. 2:**
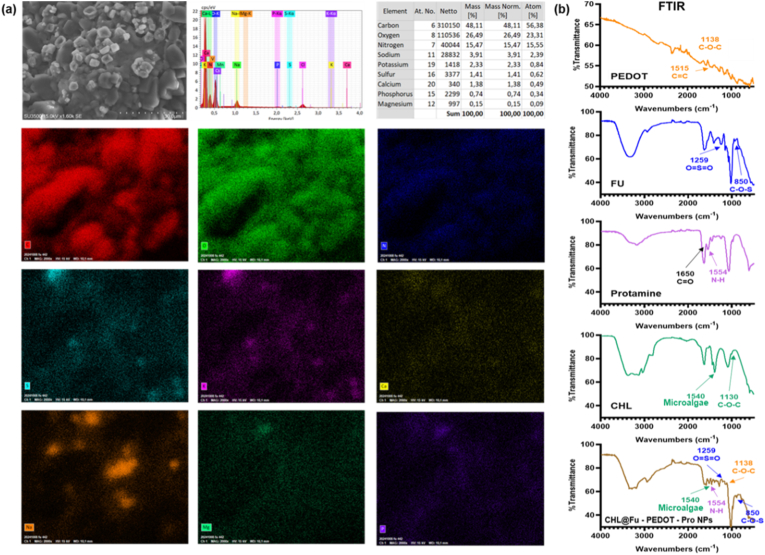
Characterization of the CHL@Pro-Fu-PEDOT-NZ system. (a) SEM image and EDS elemental mapping of CHL@Pro-Fu-PEDOT-NZs, showing the morphology and distribution of key elements such as oxygen (O), carbon (C), sulfur (S), nitrogen (N), calcium (Ca), potassium (K), magnesium (Mg), phosphorus (P), and sodium (Na). These results confirmed the successful integration of *Chlamydomonas* (CHL), fucoidan nanozymes (Fu NZs), protamine (Pro), and PEDOT into a cohesive hybrid structure. (b) FTIR spectra of CHL, Fu, Pro, PEDOT, and CHL@Pro-Fu-PEDOT-NZs. The composite spectrum reveals a combination of bands from each constituent, suggesting interactions among the components, including potential electrostatic interactions. These analyses confirmed the successful synthesis and structural integrity of CHL@Pro-Fu-PEDOT-NZs, supporting their potential as a therapeutic system.

The results provide detailed compositional, structural, and morphological insights in the CHL@Pro-Fu-PEDOT-NZ formulation, as depicted in Fig. 2. Starting with the SEM-EDS analysis shown in Fig. 2a, the complex exhibited an aggregated morphological change having a uniform distribution and distinct microscale features. SEM images indicate that the CHL@Pro-Fu-PEDOT-NZ particles were approximately 3–6 μm in size, revealing a round-shaped structure. Resultant EDS mapping confirmed the attendance of key elements including oxygen (O), carbon (C), sulfur (S), calcium (Ca), nitrogen (N), potassium (K), sodium (Na), phosphorus (P), and magnesium (Mg), in proportions that were consistent with the constituent materials: PEDOT, Pro, CHL, and Fu. The analysis for the element revealed that carbon was the predominant component at 48.11 wt%, followed by O at 26.49 wt% and N at 15.47 wt%. These results underscore the protein-rich character of Pro and the carbohydrate polymer of Fu. Additionally, the measurement of phosphorus and sulfur, among other elements, indicated the incorporation of CHL and PEDOT, suggesting the formation of a cohesive complex at the nanoscale.

#### 3.1.7 FTIR spectroscopy to verify chemical interactions and composite formation

Fig. 2b presents the FTIR spectra of CHL, Fu, Pro, PEDOT, and the hybrid CHL@Pro–Fu–PEDOT-NZs. The spectrum of CHL showed characteristic absorption bands attributable to microalgal constituents, including signals associated with chlorophyll-related functionalities and C–O–S vibrations. Fu exhibited pronounced sulfate-associated bands, consistent with its sulfated polysaccharide structure. In the Pro spectrum, prominent N–H and C=O stretching bands were observed, supporting its role as a biopolymer capable of participating in electrostatic interactions. PEDOT displayed typical conductive-polymer signatures, with strong bands at 1515 cm^−1^ (C=C stretching of the thiophene ring) and 1138 cm^−1^ (C–O–C stretching). Importantly, the CHL@Pro–Fu–PEDOT-NZs spectrum contained contributions from all constituents, confirming successful integration into a composite architecture. The emergence of additional bands and subtle peak shifts in the hybrid further suggested intercomponent interactions, most plausibly electrostatic association among the charged domains. Collectively, these results indicate that the hybrid preserves the chemical fingerprints of each component while forming an integrated structure that enables synergistic functionality.

In summary, results from the SEM-EDS, NMR, and FTIR analyses consistently supported the synthesis and characterization of the CHL@Pro-Fu-PEDOT-NZ composite. The morphology, chemical composition, and spectral profile of the composite suggested a well-structured hybrid material, with strong interactions among the components. This comprehensive analysis validated the potential of CHL@Pro-Fu-PEDOT-NZs as a promising system for following advanced biomedical applications, including DR treatment, through phototherapeutic oxygenation and enhanced photosynthetic efficiency.

### 3.2 In vitro investigational assay

#### 3.2.1 Cytocompatibility and biocompatibility of CHL-based formulations

**Fig. 3:**
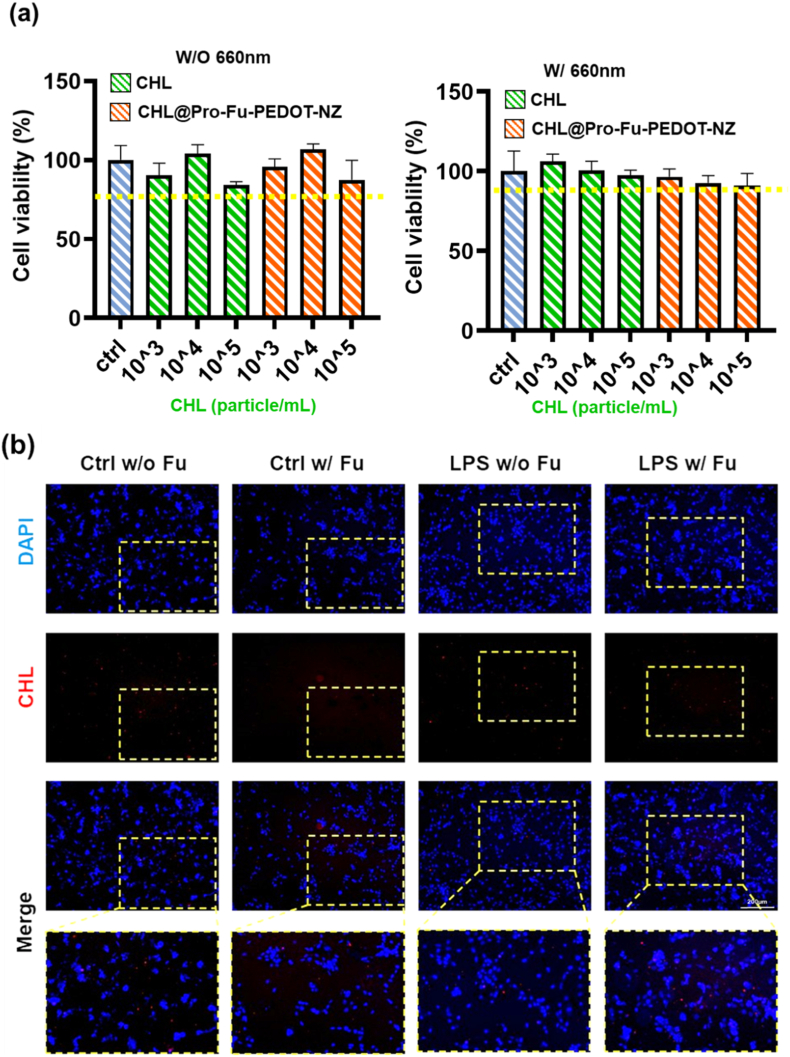
Biochemical analysis of CHL@Pro-Fu-PEDOT-NZ cytocompatibility and cellular interactions. (a) MTT assay results showing the cytotoxicity of various *Chlamydomonas* (CHL) formulations in retinal ganglion cell (RGC) cultures. Exposure to different concentrations of CHL or CHL@Pro-Fu-PEDOT-NZs, with or without 660-nm illumination, induced no significant cytotoxicity, with cell viability consistently exceeding 70%. This suggests that the synthesized phototherapeutic materials maintained excellent biocompatibility with RGCs, preserving cellular integrity and functionality. (b) Fluorescence microscopic images of RGC cultures stained with DAPI (blue) and chlorophyll (Chl, red) fluorescence, showing the localization of Chl within cells. Inflammatory stimulation with lipopolysaccharide (LPS) amplified CHL fluorescence, particularly in regions of inflammation, where increased accumulation of CHL@Pro-Fu-PEDOT-NZs was observed. Magnified images confirm the more accumulation of CHL@Pro-Fu-PEDOT-NZs in LPS-induced inflammatory regions, suggesting that the Fu component enhanced targeted delivery to inflamed eye cells via interactions with possible P-selectin or similar receptor mechanisms. These findings highlight the potential of CHL@Pro-Fu-PEDOT-NZs for targeted drug delivery and photobiomodulation therapies in treating ocular inflammation. (For interpretation of the references to colour in this figure legend, the reader is referred to the Web version of this article.)

Biochemical tests underscored the cellular interactions and cytocompatibility of the various CHL formulations. Specifically, Fig. 3a presents results of the MTT assay, revealing that exposure to various concentrations of CHL or CHL@Pro-Fu-PEDOT-NZs induced no significant cytotoxicity compared to control cells, irrespective of the application of 660-nm 660 nm illumination. Cell viability remained robust, consistently exceeding 70%, suggesting that the synthesized phototherapeutic materials maintained excellent biocompatibility with RGCs while preserving their functionality without compromising cellular proliferation or integrity. Notably, higher dosages of CHL did not have a detrimental impact on cellular growth, and the Pro-Fu-PEDOT-NZ matrix demonstrated no additional toxicity, even under photostimulation conditions (Fig. 3a). This indicated a promising potential for their use in subsequent in vivo or ex vivo ocular therapeutic applications.

#### 3.2.2 Cellular localization and inflammatory targeting behavior

The microscopic analysis revealed localization of fluorescence of Chlorophyll (depicted in red) in RGC cellular cultures, with DAPI staining highlighting nuclei in blue (Fig. 3b). Control cells without the presence of Fu (CHL@Pro-PEDOT-NZs) exhibited a relatively uniform background fluorescence. However, the introduction of Fu (CHL@Pro-Fu-PEDOT-NZ) slightly enhanced red fluorescence, indicating that the carbohydrate polymer-based Fu enabled improved retention or interactions of the component of CHL in or adjacent to the cellular surface and perinuclear areas.

Furthermore, the induction of an inflammatory condition by LPS markedly amplified CHL fluorescence near inflamed cells, particularly when Fu was incorporated into the formulation. Magnified images corroborated that CHL@Pro-Fu-PEDOT-NZs showed increased accumulation in regions displaying LPS-induced inflammation. These findings imply that potential activation of the fucoidan ligand–receptor pathway, potentially via P-selectin or related cytokine-mediated mechanisms, confers a higher binding affinity for inflamed ocular cells.

The formulation can migrate from the nasal cavity to the retina through the trigeminal nerve pathway, a mechanism currently being explored for targeted drug delivery to the posterior eye. This route bypasses biological barriers such as the blood–retinal barrier, allowing direct access to retinal tissues. The proposed process involves multiple steps: following intranasal administration, nanoparticles are absorbed through the nasal mucosa, particularly across the respiratory and olfactory epithelium. They then associate with the trigeminal nerves innervating the nasal cavity, whose ophthalmic branch extends toward ocular structures, including the cornea and eyelids. Through intra-axonal and perineural transport, the nanoparticles can move along or around the nerve fibers, eventually reaching the posterior segment of the eye, where they can deliver therapeutic agents directly to the retina. This “nose-to-retina” transport mechanism represents a promising, noninvasive alternative to conventional intravitreal injections for treating retinal diseases such as age-related macular degeneration and inherited retinal disorders.

#### 3.2.3 Potential of Fu-modified nanocarriers for inflammatory targeting

The strong correlation of intense red fluorescence nearby DAPI-counterstained cellular nuclei in these inflammatory situations highlights the potential for developing platforms aimed at targeted drug delivering and enhanced efficacy in addressing the inflammation of ocular lesions. The combination of biosafety, effectiveness, and specificity underscores a capable avenue for strategies of photobiomodulation in managing disorders of eye.

The assessment reported the high biocompatibility of the individual components PEDOT, CHL, Fu, and Pro as biomaterials, a critical factor substantiated in the literature for their use in biomedical applications [32,[60], [61], [62]]. Utilizing LPS to induce inflammation in RGCs provided a robust in vitro model that closely mimicked the inflammatory environment associated with DR, where retinal inflammation is a key driver of progression of disease [63].

#### 3.2.4 Synergistic therapeutic potential of CHL@Pro-Fu-PEDOT-NZs

Upon the inflammation situations, our results indicated that CHL@Pro-Fu-PEDOT-NZs improved red fluorescence localization signals around inflammatory cells. This suggested that the Fu component, possibly due to its bio-based characteristics and inherent receptor affinities, effectively functioned as a targeting agent, preferentially accumulating at sites of inflammation. This targeting may be mediated through interactions with pathways of P-selectin or other inflammatory cytokine-associated procedures.

The outcomes converge to indicate that the Fu NZ carrier system has significant potential for improving inflammatory therapeutic delivery to the ocular inflammation lesions. Furthermore, the existing report suggests that photostimulation can serve as a non-invasive strategy for the slowly releasing medicines from polymer carrier [[64], [65], [66], [67]].

This innovative platform integrates CHL's light-harvesting, photosynthetic machinery with PEDOT's exceptional electrical conductivity and Pro's biomolecular bridging ability, creating a powerful synergistic system. This innovative design not only preserves cellular integrity but also enhances therapeutic efficacy through localized modulation of inflammatory pathways. Such an approach presents a compelling opportunity for advancing photobiomodulation therapies, potentially yielding more-effective and minimally invasive treatments for retinal conditions associated with chronic inflammation.

#### 3.2.5 Suppression of oxidative stress via ROS scavenging in RGCs

**Fig. 4:**
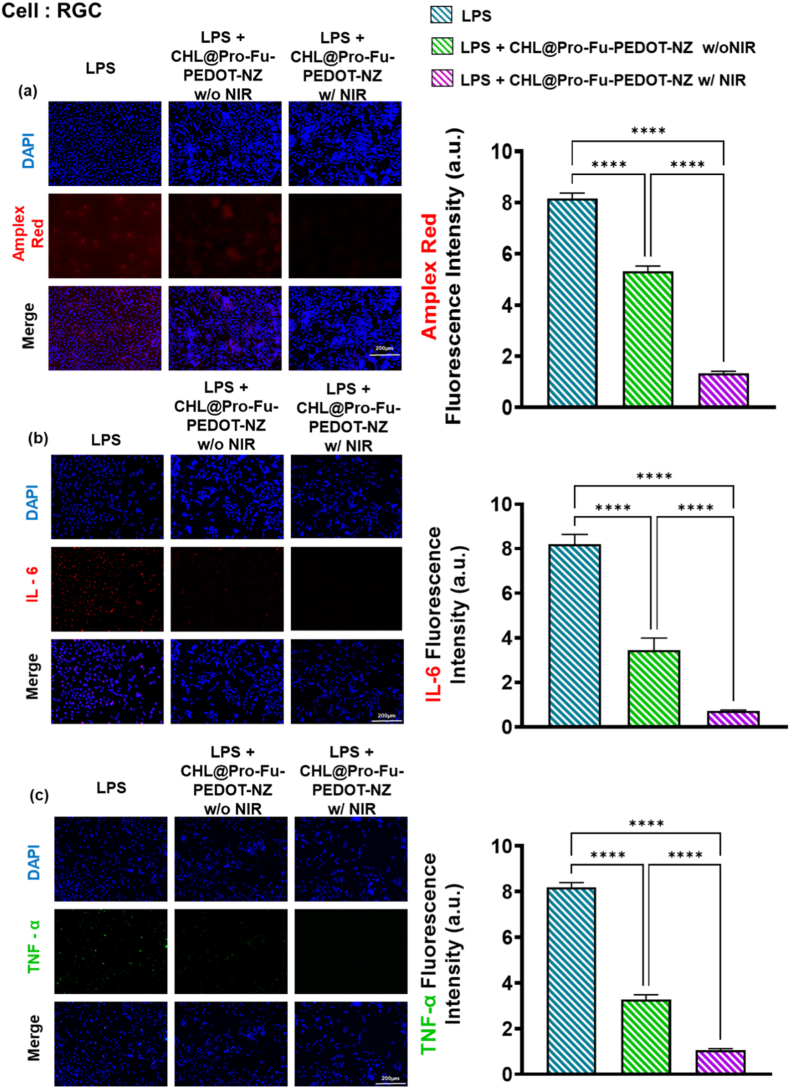
Fluorescence microscopic analysis of oxidative stress and inflammatory cytokine expression in retinal ganglion cells (RGCs) under lipopolysaccharide (LPS) stimulation and treatment with CHL@Pro-Fu-PEDOT-NZs. (a) Amplex red staining (red) quantifies intracellular reactive oxygen species (ROS) production, with DAPI staining (blue) identifying cell nuclei. The LPS-treated group showed significantly increased ROS production, while treatment with CHL@Pro-Fu-PEDOT-NZs effectively reduced the ROS intensity. The addition of 660-nm illumination further mitigated ROS levels to near the baseline, suggesting that the CHL@Pro-Fu-PEDOT-NZs provides a protective effect against oxidative stress in RGCs. (b) An IF analysis of IL-6 expression (red) demonstrates a pronounced inflammatory response in LPS-treated RGCs. Application of CHL@Pro-Fu-PEDOT-NZs reduced IL-6 levels, and 660 nm exposure further reduced IL-6 expression, indicating that the hybrid system may interfere with the LPS-induced inflammatory cascade. (c) TNF-α expression (green) followed a similar pattern, with LPS treatment causing significant inflammatory cytokine production. CHL@Pro-Fu-PEDOT-NZ treatment reduced TNF-α expression, and 660 nm irradiation further decreased TNF-α levels. These findings suggest that the combination of photosynthetic *Chlamydomonas* (CHL), conductive PEDOT, and photomodulatory 660 nm exposure effectively attenuated oxidative and inflammatory damage in RGCs, potentially restoring a near-normal physiological state. The incorporation of Fu NZs enhanced targeted delivery to inflamed cells, improving the therapeutic efficacy and minimizing off-target effects. All data are presented as mean ± standard deviation (SD). Statistical significance was defined as p < 0.05 (∗), p < 0.01 (∗∗), p < 0.001 (∗∗∗), and p < 0.0001 (∗∗∗∗). (For interpretation of the references to colour in this figure legend, the reader is referred to the Web version of this article.)

Fluorescence microscopic data we present compare those stimulated with LPS, those treated with LPS and CHL@Pro-Fu-PEDOT-NZs, and those receiving LPS plus CHL@Pro-Fu-PEDOT-NZs upon 660 nm treatment (Fig. 4). Fig. 4a visualizes intracellular ROS with Amplex Red (red) and marks nuclei with DAPI (blue). Retinal ganglion cells exposed to LPS show a high level in red fluorescence, indicating a substantial rise in oxidative stress. Treatment with CHL@Pro-Fu-PEDOT-NZs effectively mitigated this fluorescence intensity, and the application of 660-nm irradiation further decreased ROS levels to near the baseline, indicating that the photoelectrical or photo properties of the CHL@Pro-Fu-PEDOT-NZs-constructed complex provided a protective effect against oxidative damage in RGCs.

#### 3.2.6 Inhibition of IL-6-mediated inflammatory response

A cellular assay examined expression of the inflammatory cytokine IL-6 utilizing a red IF label (Fig. 4b). The LPS-treated cohort exhibited pronounced IL-6 activation, indicating a significant inflammatory response. Notably, the application of CHL@Pro-Fu-PEDOT-NZs resulted in a marked decrease in the IL-6 signal intensity. Furthermore, the 660 nm–irradiated CHL@Pro-Fu-PEDOT-NZ group exhibited an even greater reduction in IL-6 levels. These findings implied that the hybrid system may effectively interfere with or attenuate the LPS-induced inflammatory cascade, which characteristically heightens IL-6 production in stressed RGCs when activated by the appropriate wavelength of light.

#### 3.2.7 Reduction of TNF-α expression through photomodulation

Fig. 4c illustrates a comparable pattern in TNF‐α expression (green), a cytokine associated with inflammatory retina. The fluorescent intensity in the LPS-only group indicates a considerable response to inflammation. However, the group given CHL@Pro-Fu-PEDOT-NZs effectively diminished the fluorescent signal, and the treatment of additional 660 nm reduced further TNF‐α levels. This synergistic interaction between the CHL-based photosensitizer, the Pro-Fu-PEDOT-NZ matrix, and 660 nm exposure likely enhanced electron transfer or induced a photomodulatory effect, thereby curbing the release of inflammatory mediators.

#### 3.2.8 Mechanistic insight: photosynthesis-driven inflammation modulation

These findings suggest that integrating photosynthetic or photochemical components into a purposefully engineered nano formulation may offer a powerful strategy to protect retinal ganglion cells (RGCs) against LPS-induced damage. Lipopolysaccharide (LPS) exposure is well known to trigger a significant increase in proinflammatory cytokines, including TNF-α and IL-6, as well as elevated intracellular reactive oxygen species (ROS) levels [68], a response clearly illustrated in the cell experiments [69]. In contrast, the recent literature has elucidated the potent antioxidant and anti-inflammatory properties conferred by the formation of oxygen via photosynthesis [[70], [71], [72]].

Within this design, the CHL@Pro-Fu-PEDOT-NZ composite integrates the matrix's light-harvesting capability with the intrinsic conductivity of PEDOT along with Fu NZ, possibly facilitating rapid electron transfer and thereby eliciting beneficial biological effects.

#### 3.2.9 Reprogramming macrophage polarization under phototherapeutic stimulation

**Fig. 5:**
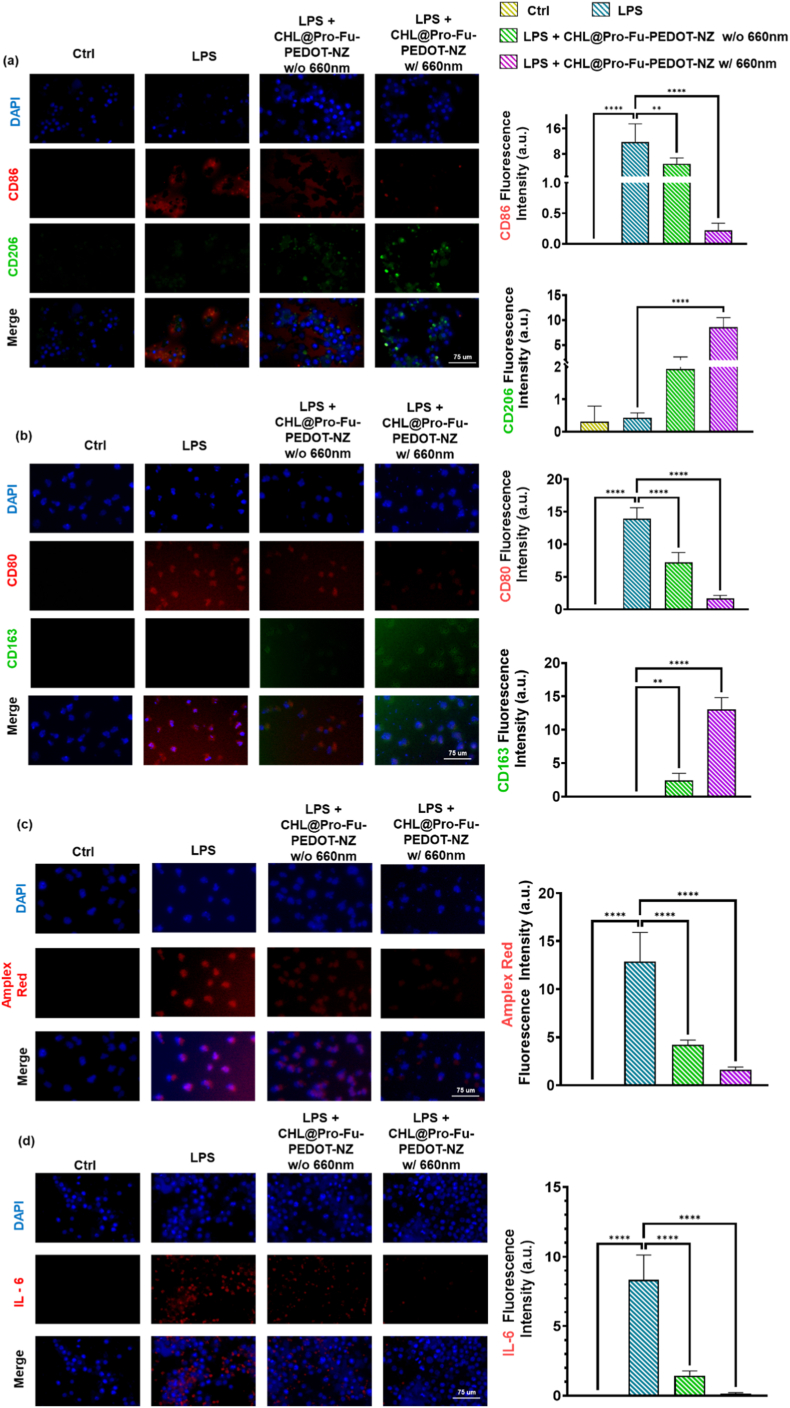
Modulation of macrophage polarization and inflammatory responses by CHL@Pro-Fu-PEDOT-NZs under lipopolysaccharide (LPS) stimulation and 660 nm exposure. (a) Fluorescence imaging of LPS-treated RAW macrophages shows a marked shift towards a proinflammatory M1 (CD86) phenotype, characterized by minimal green-labeled M2 (CD206) signals and increased red-labeled M1 polarization. The group given CHL@Pro-Fu-PEDOT-NZs effectively reversed this trend, with subsequent 660-nm light exposure enhancing the M2 population. (b)Immunofluorescence staining of macrophage polarization markers CD80 (M1) and CD163 (M2). Control macrophages showed low baseline expression of both markers. LPS stimulation significantly increased CD80 expression with weak CD163, confirming M1 polarization. Treatment with LPS + CHL@Pro-Fu-PEDOT-NZ (without 660 nm) reduced CD80 and moderately increased CD163. Notably, LPS + CHL@Pro-Fu-PEDOT-NZ with 660 nm irradiation produced the strongest shift toward the M2 phenotype, characterized by decreased CD80 and increased CD163 expression. Quantification confirmed reduced M1 marker levels and enhanced M2 marker levels, with the most significant effect observed under 660 nm activation. (c) An Amplex red assay quantifies intracellular reactive oxygen species (ROS), revealing an increase in ROS levels in LPS-treated macrophages. (d) IF analysis of IL-6 expression shows significantly elevated IL-6 levels in LPS-only macrophages, which notably decreased upon treatment with CHL@Pro-Fu-PEDOT-NZs. A further reduction was observed with 660 nm exposure. These findings highlight the effectiveness of the CHL@Pro-Fu-PEDOT-NZ phototherapeutic biohybrid in mitigating LPS-induced macrophage activation, promoting a shift towards the anti-inflammatory M2 phenotype. The photochemical properties of the system, including enhanced electron transfer and 660 nm activation, likely contributed to this immunomodulatory effect. This integrated approach, combining chlorophyll's light-harvesting capabilities, the conductivity of PEDOT, and fucoidan nanozyme (Fu NZ)'s targeting ability, offers a promising strategy for immunomodulation treatments heading for reconditioning homeostasis of tissue in inflammatory diseases. All data are presented as mean ± standard deviation (SD). Statistical significance was defined as p < 0.05 (∗), p < 0.01 (∗∗), p < 0.001 (∗∗∗), and p < 0.0001 (∗∗∗∗). (For interpretation of the references to colour in this figure legend, the reader is referred to the Web version of this article.)

As illustrated in Fig. 5, LPS-pretreated RAW macrophage cells exhibited a transition from an inflammatory phenotype to a more homeostatic situation in the presence of CHL@Pro-Fu-PEDOT-NZs upon 660-nm light exposure. Immunofluorescence staining was used to evaluate macrophage polarization under different treatments (Fig. 5a and b). In the control group, macrophages showed low baseline expression of M1 markers (CD86/CD80, red) and limited M2 marker expression (CD206/CD163, green). Upon LPS stimulation, macrophages exhibited a marked increase in CD86 and CD80 signals with comparatively weak CD206/CD163, confirming successful induction of a pro-inflammatory M1 phenotype. Treatment with LPS + CHL@Pro-Fu-PEDOT-NZ (w/o 660 nm) reduced the M1-associated fluorescence intensity and moderately enhanced M2-associated markers, indicating partial repolarization. Notably, LPS + CHL@Pro-Fu-PEDOT-NZ (w/660 nm) produced the strongest phenotypic shift, characterized by suppressed CD86/CD80 and prominently increased CD206/CD163 staining. Quantification (right panels) corroborated these trends, showing decreased M1 marker levels and elevated M2 marker levels in the treatment groups, with the most pronounced effect observed under 660 nm activation.

#### 3.2.10 Suppression of oxidative stress and IL-6 secretion in macrophages

A similar pattern emerged in the Amplex Red assay (Fig. 5c): LPS exposure drove intracellular ROS to multiple times the baseline level. Administration of CHL@Pro-Fu-PEDOT-NZs reduced this ROS surge by approximately 50%, and with 660 nm illumination, ROS levels were further restored to near-normal levels. Moreover, the IL-6 IF analysis (Fig. 5d) showed elevated IL-6 levels in the LPS-only group, which significantly decreased with the addition of CHL@Pro-Fu-PEDOT-NZs, and an even more substantial reduction was observed in samples further subjected to 660 nm irradiation.

#### 3.2.11 Mechanistic insight into immunomodulation by phototherapeutic biohybrids

The simultaneous decline in pro-inflammatory M1 markers, intracellular ROS, and IL-6 secretion demonstrates that the CHL@Pro-Fu-PEDOT-NZ biohybrid effectively blunts LPS-driven macrophage activation. These benefits probably stem from the material's ability—under 660 nm illumination—to accelerate electron transfer and photochemical reactions, thereby dampening inflammatory signaling and steering macrophages toward an M2, tissue-repair phenotype.

#### 3.2.12 Conceptualizing CHL as a nanoscale immunomodulatory microalgae

LPS is a well-established trigger of M1 macrophage polarization, driving a pro-inflammatory phenotype characterized by heightened cytokine release and oxidative stress. Our findings show that integrating CHL, PEDOT, Pro, and Fu NZ into a single phototherapeutic biohybrid markedly tempers this response, steering macrophages toward the reparative M2 state instead. This shift is consistent with earlier reports that photosynthetically generated therapeutic gases can quell inflammation and reprogramme macrophages from a harmful M1 profile to an anti-inflammatory, tissue-healing M2 phenotype [71]. In this framework, the Fu NZs acted not only as a lesion-targeting agent with intrinsic capabilities of homing to inflamed tissues but also as a bio-mediator that, in conjunction with the bioelectronic characteristics of PEDOT, helped establish a more beneficial immunomodulatory microenvironment.

### 3.3 In vivo experiments

#### 3.3.1 In vivo biodistribution and targeted ocular delivery via intranasal administration

To evaluate the biodistribution, delivery efficiency, and biosafety of pure CHL (live or dead) administered via different routes, we first performed in vivo/ex vivo fluorescence imaging, apoptotic analysis, and histological assessments.

As shown in Fig. S2a, in vivo and ex vivo fluorescence imaging revealed that only the intranasal delivery (IND) of live CHL resulted in a strong and localized fluorescence signal in the ocular region, whereas IND (dead), intraperitoneal (IP), and topical (OP) groups exhibited negligible or no detectable signal. This finding highlights that both the delivery route and microalgal viability are critical determinants of retinal accumulation efficiency. The absence of fluorescence signals in major organs further indicates minimal systemic distribution and off-target accumulation, supporting the localized and non-invasive nature of IND-mediated delivery. This phenomenon may be partially attributed to the intrinsic phototactic behavior of CHL; however, given that no controlled 660 nm irradiation was applied, ambient light exposure may only provide a limited guiding effect. Alternatively, the enhanced retinal accumulation observed in the IND (live) group may also arise from improved mucosal penetration and active motility of viable CHL. Quantitative analysis further confirmed significantly higher fluorescence intensity in the IND (live) group compared to all other conditions, reinforcing its superior delivery performance. Consistently, TUNEL staining (Fig. S2b) demonstrated a less apoptotic signals in the IND (live) group, suggesting that effective retinal delivery alleviates without serious apoptosis.

Furthermore, histological evaluation of major organs (heart, liver, spleen, lung, kidney, and eye), as presented in Fig. S3, demonstrated that all treatment (CHL) groups without observable signs of necrosis, inflammatory infiltration, fibrosis, or structural abnormalities. These results indicate that CHL administration, regardless of microalgal viability or delivery route, does not induce detectable systemic toxicity or organ damage. Collectively, these findings demonstrate that IND of live CHL enables efficient and cause retinal accumulation while minimizing off-target effects and systemic toxicity. This favorable balance between delivery efficacy and biosafety underscores the translational potential of this platform as a non-invasive therapeutic strategy for retinal diseases such as diabetic retinopathy.

**Fig. 6:**
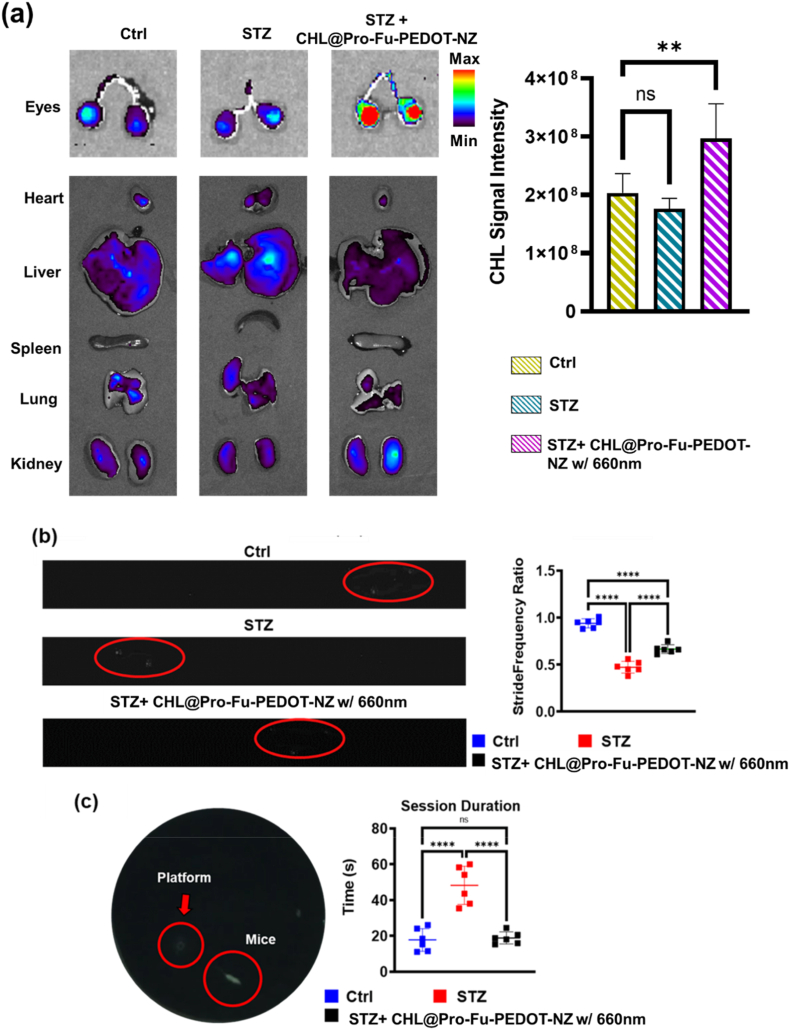
In vivo evaluation of CHL@Pro-Fu-PEDOT-NZs in mice with STZ-induced diabetic retinopathy (DR). (a) In vivo imaging of Chlorophyll fluorescence following intranasal administration of CHL@Pro-Fu-PEDOT-NZs, showing significant fluorescence localized in the ocular region under 660-nm illumination. Fluorescence images were acquired 24 h after intranasal administration of CHL@Pro-Fu-PEDOT-NZs using an in vivo imaging system under 660 nm excitation. In contrast, control mice and untreated STZ-administered animals exhibited only baseline autofluorescence, demonstrating effective targeting and translocation of the biohybrid complex to the retina. (b) Gait analysis. (c) Swimming performance from the water maze test showing that STZ mice treated with CHL@Pro-Fu-PEDOT-NZs under 660 nm irradiation. These findings highlight the therapeutic potential of CHL@Pro-Fu-PEDOT-NZs in improving behavior through a light-driven biohybrid approach, utilizing intranasal administration and targeted ocular delivery. All data are presented as mean ± standard deviation (SD). Statistical significance was defined as p < 0.05 (∗), p < 0.01 (∗∗), p < 0.001 (∗∗∗), and p < 0.0001 (∗∗∗∗).

Building on the auguring cellular and above findings, we undertook a comprehensive in vivo assessment to evaluate the efficacy and safety of CHL@Pro-Fu-PEDOT-NZs. Utilizing a murine model of STZ-created DR, we aimed to confirm the therapeutic aptitude and formulation biocompatibility. In vivo imaging revealed a significant CHL fluorescence signal localized in the ocular region of mice with STZ-induced DR, administered CHL@Pro-Fu-PEDOT-NZs via the nasal route, as illustrated in Fig. 6a. Control or STZ-given animals displayed only the expected baseline autofluorescence. By contrast, the treated group exhibited a pronounced fluorescence increase, suggesting that the biohybrid complex efficiently migrated from the nasal cavity to the retina (Fig. 6a). This targeted delivery is expected to substantially boost therapeutic efficacy.

#### 3.3.2 Improvements in locomotion and cognitive performance in treated diabetic mice

The gait analysis results (Fig. 6b) showed that STZ-treated mice exhibited a reduced stride frequency compared with the control group. In contrast, mice receiving CHL@Pro–Fu–PEDOT-NZs with 660-nm irradiation displayed stride frequencies approaching to controls, suggesting an improvement in STZ-associated functional impairment and overall alleviation of diabetic retinopathy–related deficits.

Similarly, swimming performance data from the water maze (Fig. 6c) reinforced these findings: STZ mice administered CHL@Pro-Fu-PEDOT-NZs upon 660 nm treatment demonstrated platform localization latencies comparable to those of control subjects, while the untreated STZ-given group showed significantly prolonged search durations. This therapeutic efficacy likely stemmed from synergistic mechanisms involving Chl's photosensitizing properties, PEDOT's conductive characteristics, and the targeted delivery facilitated by polysaccharides Fu, resulting in reduced DR stress and its systemic ramifications. These results indicate a promising therapeutic avenue for addressing DR pathology and its functional impairments through a nasal-to-eye administration strategy that capitalizes on light-driven biohybrid interactions.

##### 3.3.2.1 Histological assessment of retinal ROS reduction and anti-inflammatory effects

**Fig. 7:**
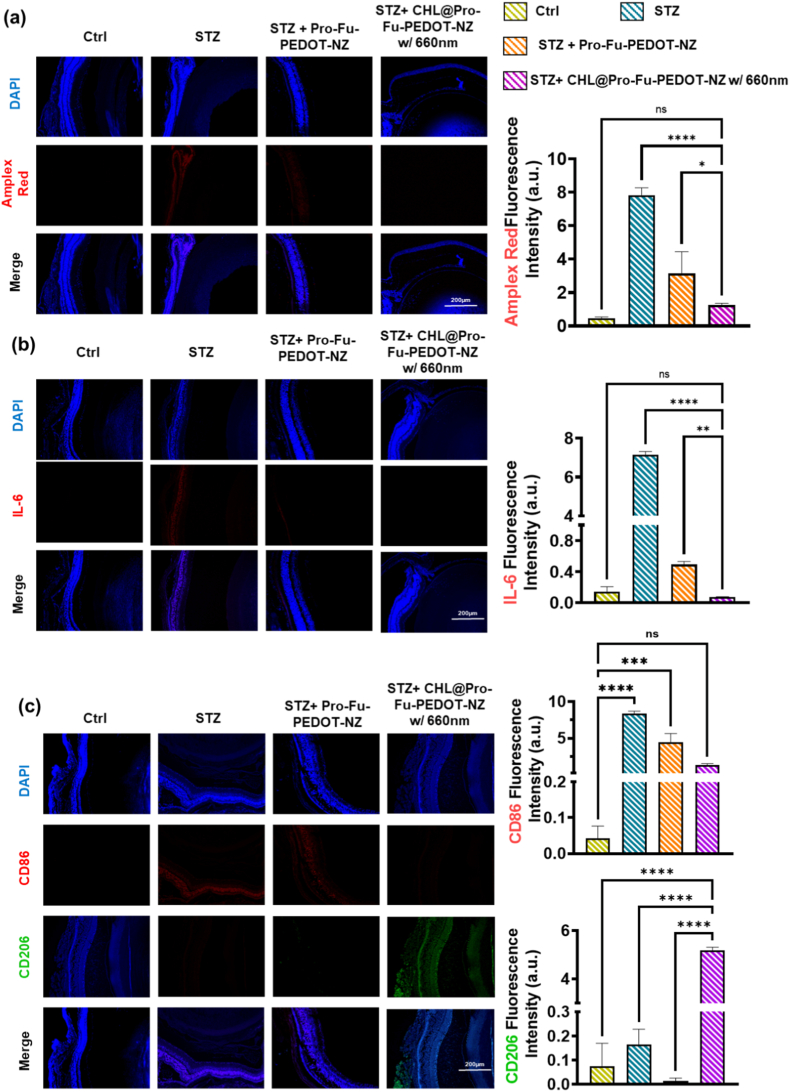
**Histological analysis of ocular tissue sections from mice with STZ-stimulated diabetic retinopathy (DR) given with CHL@Pro-Fu-PEDOT-NZs and activated by 660-nm light.** (a) Amplex red fluorescence indicates reactive oxygen species (ROS) accumulation in ocular tissue sections. The treatment cohort showed a significant reduction in ROS levels, approaching those observed in normal controls, highlighting the antioxidant effects of CHL@Pro-Fu-PEDOT-NZs. (b) IF analysis of interleukin (IL)-6 signaling, showing a marked decrease in IL-6 expression in CHL@Pro-Fu-PEDOT-NZs-treated samples, particularly under 660 nm activation, indicating reduced inflammation following treatment. (c) Macrophage polarization analysis using M1 (CD86 red) and M2 (CD206 green) markers. In the STZ group, there was a predominance of M1 markers, which significantly decreased after treatment, while M2 markers increased, suggesting a shift from a proinflammatory to a reparative macrophage phenotype. These findings demonstrated that CHL@Pro-Fu-PEDOT-NZs effectively mitigated oxidative stress, reduced inflammation, and reorganized the macrophage profile towards a healthier, reparative state in diabetic retinal tissues. The results underscore the therapeutic potential of combining photosynthetic electron transfer with targeted delivery of Fu NZs to enhance tissue repair in inflammatory conditions like diabetic retinopathy. All data are presented as mean ± standard deviation (SD). Statistical significance was defined as p < 0.05 (∗), p < 0.01 (∗∗), p < 0.001 (∗∗∗), and p < 0.0001 (∗∗∗∗). (For interpretation of the references to colour in this figure legend, the reader is referred to the Web version of this article.)

Histological examination of retinal sections underscores the therapeutic benefit of intranasally delivered, 660 nm-activated CHL@Pro-Fu-PEDOT-NZs in the STZ-induced diabetic-retinopathy model. In Fig. 7a, Amplex Red fluorescence, which reflects intracellular ROS levels, drops sharply in treated eyes, approaching the low baseline observed in healthy controls and contrasting starkly with the intense signal seen in untreated STZ mice Fig. 7(a). Fig. 7b further illustrates this effect, showing a substantial decrease in IL-6 signaling following treatment with the CHL@Pro-Fu-PEDOT-NZ formulation, particularly under 660 nm activation.

##### 3.3.2.2 Macrophage polarization shift toward reparative phenotype in retinal tissues

Additionally, Fig. 7c reveals a notable shift in macrophage polarization; the heightened M1 marker (red) found in the STZ group significantly diminished post-treatment, while the M2 marker (green) increased, indicating that photochemically enhanced electron transfer may attenuate proinflammatory pathways and promote a reparative macrophage phenotype. These findings collectively demonstrated that the targeted delivery of CHL via Pro-Fu-PEDOT-NZs effectively mitigated oxidative stress and inflammation, while also reorganizing the macrophage profile in diabetic retinal tissue towards a healthier state.

##### 3.3.2.3 Synergistic therapeutic mechanisms: photochemical gas production and inflammation targeting

The existing literature highlights the electrochemical bioactivity from processes of photosynthesis [73], especially the formation of oxygen, and their efficacy in mitigating oxidative stress and cellular inflammation. In the proposed approach, the increased generation of these therapeutic gases, coupled with the lesion-targeting competencies of polymeric Fu, established a double means that not only scavenged the ROS but also dampened inflammation.

##### 3.3.2.4 Immunofluorescence analysis of hypoxia and vascularization biomarkers in retinal tissue

**Fig. 8:**
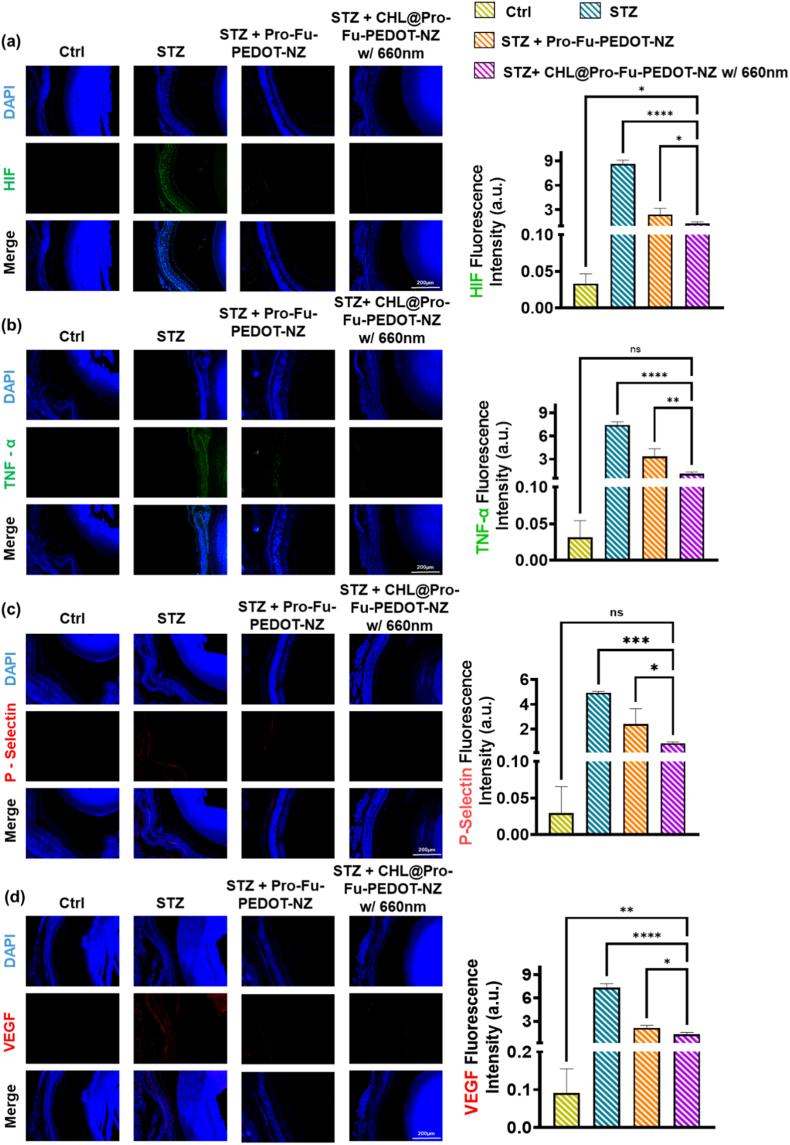
Immunofluorescence staining of critical ocular biomarkers was performed on groups: healthy mice, mice with STZ-induced diabetic retinopathy, STZ-induced diabetic retinopathy mice treated with CHL@Pro-Fu-PEDOT-NZs, and STZ-induced diabetic retinopathy mice treated with CHL@Pro-Fu-PEDOT-NZs under 660-nm near-infrared irradiation. (a) HIF-1α expression (green), a marker of hypoxia, prominently increased in the untreated STZ group, while the treated cohort exhibited fluorescence levels similar to the normal control, indicating improved oxygen homeostasis post-phototherapy. (b) TNF-α expression (green), a proinflammatory cytokine, was significantly elevated in untreated diabetic tissues but was markedly reduced following treatment with CHL@Pro-Fu-PEDOT-NZs and 660 nm exposure, suggesting an anti-inflammatory effect. (c) P-selectin expression (red), associated with leukocyte recruitment and inflammation, was notably decreased in the treatment group compared to the STZ-only group, highlighting the biohybrid's impact on inflammation. (d) Vascular endothelial growth factor (VEGF) expression (red), a key factor in pathological neovascularization, showed a significant reduction in the treated cohort, aligning more closely with the healthy control tissue profile. These results demonstrate that nasal administration of CHL@Pro-Fu-PEDOT-NZs, combined with 660 nm light, ameliorated hypoxia, reduced inflammation, and modulated abnormal angiogenesis in DR via improved electron transfer and photochemically-driven modulation of illness-associated pathways. All data are presented as mean ± standard deviation (SD). Statistical significance was defined as p < 0.05 (∗), p < 0.01 (∗∗), p < 0.001 (∗∗∗), and p < 0.0001 (∗∗∗∗). (For interpretation of the references to colour in this figure legend, the reader is referred to the Web version of this article.)

Fig. 8 summarizes immunofluorescence staining of key retinal biomarkers in groups: healthy controls, STZ-induced diabetic-retinopathy (DR) mice, and DR mice treated with CHL@Pro-Fu-PEDOT-NZs under 660-nm near-infrared light. In Fig. 8a, immunofluorescence staining for HIF-1α revealed less signal in the control group, indicating normoxic tissue conditions. In contrast, the STZ group exhibited a marked increase in HIF-1α fluorescence intensity, particularly within the affected tissue regions, consistent with hypoxia-associated stress induced by STZ. Treatment with Pro-Fu-PEDOT-NZs resulted in a noticeable reduction in HIF-1α expression compared with the STZ group, suggesting partial alleviation of hypoxic conditions. Notably, the STZ + CHL@Pro-Fu-PEDOT-NZs w/660 nm group showed the lowest HIF-1α signal among the treated groups, approaching levels observed in the control, indicating effective mitigation of hypoxia-related stress under 660 nm activation. Quantitative analysis confirmed a stepwise decrease in HIF-1α intensity from STZ to treatment groups. This suggests an improvement in oxygen homeostasis post-phototherapy. A similar pattern is evident in Fig. 8b for TNF-α which showed weak basal expression in the Control group, consistent with a non-inflammatory state. The STZ group displayed strong TNF-α fluorescence, indicating a pronounced pro-inflammatory microenvironment. Following treatment with Pro-Fu-PEDOT-NZs, TNF-α expression was reduced, reflecting attenuation of inflammatory signaling. The STZ + CHL@Pro-Fu-PEDOT-NZs w/660 nm group demonstrated the most pronounced suppression of TNF-α expression, with fluorescence intensity markedly lower than that of the STZ group. Quantitative results supported these observations, confirming effective inflammation modulation in the treated groups.

##### 3.3.2.5 Mitigation of inflammation, hypoxia, and pathological angiogenesis via CHL@Pro-Fu-PEDOT-NZs treatment

Immunofluorescence analysis of P-selectin (Fig. 8c) revealed minimal expression in the control group, indicating intact endothelial homeostasis. In contrast, the STZ group exhibited elevated P-selectin staining, suggesting endothelial activation and increased leukocyte adhesion potential. Treatment with Pro-Fu-PEDOT-NZs resulted in reduced P-selectin expression relative to the STZ group, indicating partial restoration of endothelial function. The STZ + CHL@Pro-Fu-PEDOT-NZs w/660 nm group showed minimized expression of P-selectin fluorescence, approaching control levels, suggesting attuned vascular activity and reduced inflammatory endothelial activation. These trends were corroborated by quantitative fluorescence analysis. Additionally, panel Fig. 8d addresses VEGF (red), a crucial factor linked to pathological neovascularization. VEGF staining in the control group showed low basal expression, consistent with normal tissue homeostasis. The STZ group exhibited moderately increased VEGF levels, likely reflecting a compensatory response to tissue injury. Treatment with Pro-Fu-PEDOT-NZs significantly reduced VEGF expression compared with both the control and STZ groups, indicating alteration of pro-angiogenic signaling pathways. Importantly, the STZ + CHL@Pro-Fu-PEDOT-NZs w/660 nm group demonstrated the lowest to no VEGF fluorescence intensity, suggesting that 660 nm-assisted treatment further inhibit angiogenic activity. Taken together, our results indicate that intranasal delivery of CHL@Pro-Fu-PEDOT-NZs, activated by 660 nm irradiation, alleviates retinal hypoxia, vascularization and inflammation and simultaneously normalizes pathological angiogenesis in diabetic retinopathy. These therapeutic benefits appear to stem from intensified electron transfer and light-driven modulation of critical disease-associated signaling pathways.

Oxygen generated through photosynthesis critically alleviates hypoxic microenvironments, as highlighted in the literature [74]. In the context of DR, tissue hypoxia is a significant contributor to pathological neovascularization. Enhancing oxygen homeostasis can effectively reduce this aberrant vascular proliferation [75].

Data presented in Fig. 8 demonstrate that the application of CHL@Pro-Fu-PEDOT-NZs under 660-nm illumination led to marked decreases in expressions of hypoxia markers, such as HIF-1α, and proinflammatory cytokines, including TNF-α. Additionally, there was a significant reduction in signals associated with the VEGF angiogenic factor and P-selectin (leukocyte recruitment). These findings are consistent with existing research that highlights the ability of photosynthesis to mitigate oxidative stress and inflammation.

Moreover, integration of Fu NZs facilitated the targeted localization of the biohybrid at inflammatory lesions, concentrating the therapeutic effects in areas most in need of oxygen restoration. This synergistic mechanism not only normalized the retinal microenvironment by attenuating hypoxia-driven signaling pathways but also contributed to the reduction of pathological neovascularization. Looking ahead, it is plausible that these advanced, light-responsive biohybrid systems could function akin to autonomous therapeutic agents, dynamically modulating cellular signaling pathways to achieve tissue homeostasis with precision in treating DR and other conditions associated with hypoxia.

##### 3.3.2.6 Histopathological evaluation of systemic and ocular tissues following CHL@Pro-Fu-PEDOT-NZs treatment in diabetic retinopathy mice

**Fig. 9:**
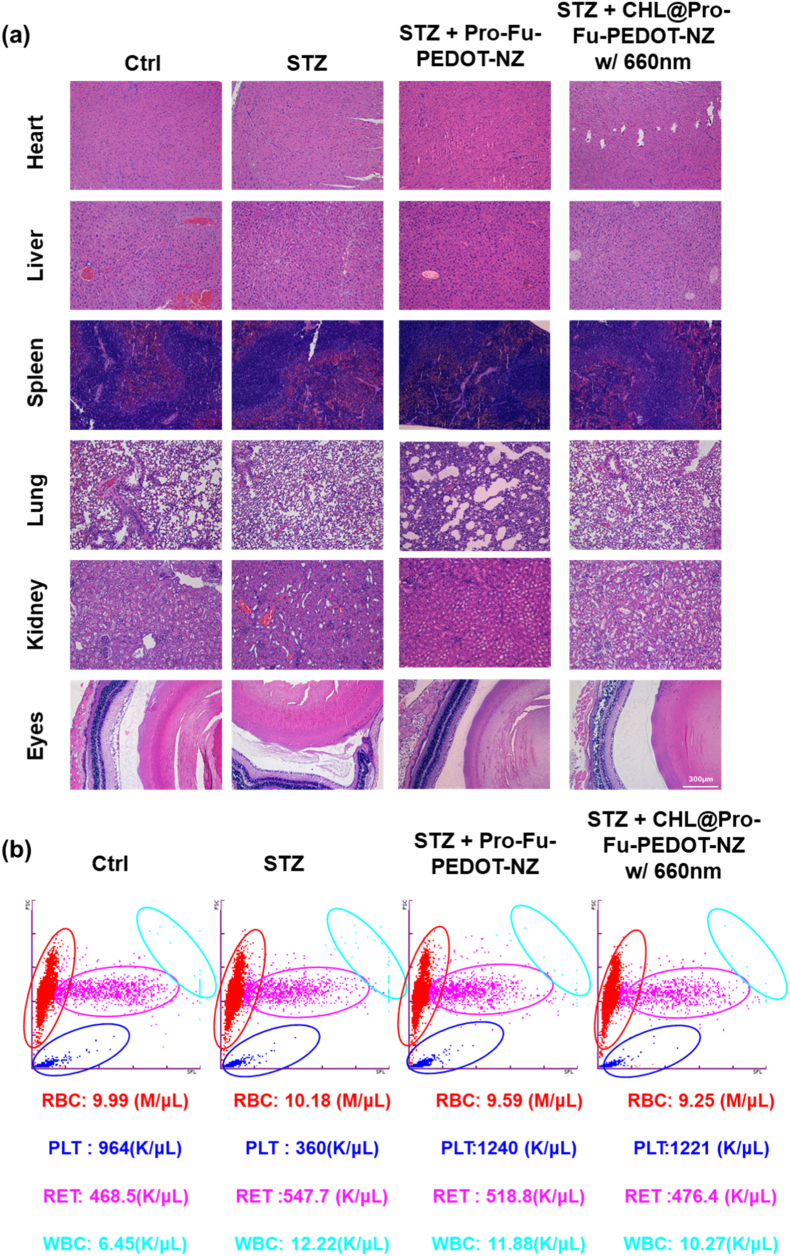
**In vivo biocompatibility assessment of intranasally administered Pro–Fu–PEDOT-NZs and CHL@Pro–Fu–PEDOT-NZs with 660-nm irradiation in the STZ-induced DR model.** (a) Representative H&E-stained sections of major organs (heart, liver, spleen, lung, and kidney) and ocular tissues from the indicated groups, showing preserved tissue architecture without detectable pathological abnormalities under the tested conditions. (b) Flow cytometric hematological scatter profiles illustrating distributions of major blood cell populations, including red blood cells (RBCs), platelets (PLTs), reticulocytes (RETs), and white blood cells (WBCs), across all groups. Control groups included STZ, STZ + Pro–Fu–PEDOT-NZs (CHL-free control), and STZ + CHL@Pro–Fu–PEDOT-NZs with 660-nm light exposure, enabling evaluation of systemic safety and component-level biocompatibility. (For interpretation of the references to colour in this figure legend, the reader is referred to the Web version of this article.)

Regarding long-term biocompatibility, we agree that while the current study confirms safety through histopathology (Fig. 9a) and hematological profiling (Fig. 9b), the immune response following repeated intranasal administration of an algae-based system warrants further consideration. Although no apparent organ toxicity or abnormal blood cell distributions were observed under the present dosing schedule, algal-derived components and hybrid nanocomposites may still pose potential risks of cumulative immunogenicity (e.g., mucosal immune activation, anti-algal antibody formation) or chronic inflammation after prolonged exposure. We have therefore added a discussion acknowledging this limitation and outlining ongoing/future plans to evaluate long-term safety, including repeated-dose studies over extended periods with longitudinal monitoring of systemic inflammatory cytokines, anti-CHL/anti-component antibody responses (IgG/IgA), complement activation, and detailed histological assessment of nasal mucosa and major organs.

Consistently, the hematological flow cytometry scatter profiles (Fig. 9b) showed well-resolved and comparable distributions of major blood cell populations, including RBCs, PLTs, RETs, and WBCs, across all groups. No distorted scatter patterns, abnormal population overlap, or features suggestive of hematological toxicity were observed. Together, these data indicate that intranasal administration of Pro-Fu-PEDOT-NZs or CHL@Pro-Fu-PEDOT-NZs with 660-nm light exposure does not measurably disrupt systemic blood homeostasis or induce detectable organ toxicity in the STZ-induced DR model.

Western blot analysis for the treated eye tissues (Fig. S4) showed low basal expression of IL-6 in the Control group. In the STZ group, IL-6 expression was markedly increased, indicating activation of disease-associated signaling pathways. Treatment with Pro-Fu-PEDOT-NZs resulted in a clear reduction in IL-6 expression compared with the STZ group, suggesting partial suppression of pathological signaling. Notably, the CHL@Pro-Fu-PEDOT-NZs with 660 nm group exhibited a further decrease in IL-6 levels, approaching those observed in the control group. Densitometric analysis confirmed a downregulation of IL-6 in the treated groups relative to the STZ group. Quantitative analysis of IL-6 protein expression, normalized to GAPDH and expressed relative to the control group, revealed a marked increase in the STZ group (274.1%) compared with the control (100%). Treatment with Pro-Fu-PEDOT-NZs significantly reduced IL-6 levels to about 131.4%, while the STZ + CHL@Pro-Fu-PEDOT-NZs with 660 nm group showed a further decrease to around 111.5%, approaching baseline values. These results indicate effective attenuation of STZ-induced inflammatory signaling, with the 660 nm-activated CHL-loaded formulation demonstrating the strongest suppressive effect on IL-6 expression.

Expression of TNF-α followed a similar trend. Minimal expression was detected in the control group, whereas the STZ group displayed a pronounced increase in TNF-α levels. Administration of Pro-Fu-PEDOT-NZs significantly attenuated TNF-α expression, while CHL@Pro-Fu-PEDOT-NZs with 660 nm resulted in the most substantial suppression. Quantitative band intensity analysis supported these observations, demonstrating effective modulation of TNF-α expression by the treatment, particularly under 660 nm activation. Quantitative analysis of TNF-α protein expression, normalized to GAPDH and expressed relative to the control group, showed a substantial increase in the STZ group (around 220.2%) compared with the control (100%). Treatment with Pro-Fu-PEDOT-NZs markedly reduced TNF-α levels to around 137.4%, indicating partial suppression of STZ-induced inflammation. Notably, the STZ + CHL@Pro-Fu-PEDOT-NZs with 660 nm group further decreased TNF-α expression to 97.3%, returning to near-baseline levels. These results demonstrate effective attenuation of TNF-α–mediated inflammatory responses, with the 660 nm-activated CHL-loaded formulation showing the strongest inhibitory effect.

## 4 Discussion

The present study demonstrates that the CHL@Pro-Fu-PEDOT-NZs system integrates photochemical, photosynthesis, and biological functionalities to provide a multifaceted therapeutic platform for DR. Unlike traditional anti-VEGF or corticosteroid-based approaches, this biohybrid platform combines photosynthetic electron transfer and therapeutic oxygen with 660 nm-responsive activation, enabling simultaneous modulation of vascularization and hypoxia. The suggested potential improvements in retinal function and tissue protection align with previous studies reporting the efficacy of PEDOT-based photothermal systems and Fucoidan-mediated retinal targeting for ocular therapy. The enhanced localization and bioavailability achieved through nasal delivery further support the potential of the nose-to-retina transport route, which has been previously described as a noninvasive alternative to intravitreal injections. Beyond its photochemical role, CHL may also aid spatial targeting via light-guided motility. Local retinal illumination could provide a directional cue that should promote migration of CHL toward the irradiated area, while PEDOT enhances internal light conduction within the hybrid structure, together facilitating phototaxis-driven enrichment at the target tissue.

Upon 660 nm irradiation, the CHL@Pro-Fu-PEDOT-NZs system undergoes photochemical activation, generating bioactive gases such as O_2_ that contribute to redox balance and tissue protection in the diabetic retina. The released O_2_ enhances local oxygenation, supports mitochondrial function, and mitigates excessive ROS accumulation, thereby restoring oxidative homeostasis. In addition, based on prior evidence, electrical stimulation also enhances antioxidants. Although this mechanism was not directly examined in the present study, such antioxidative interactions may partially contribute to the observed reduction in oxidative damage. Previous reports further link these effects to activation of redox-regulatory pathways such as Nrf2/HO-1, enhancing endogenous antioxidant defenses. While inflammatory and angiogenic markers were not assessed, the improved tissue morphology and oxidative status observed here suggest that the therapeutic efficacy of CHL@Pro-Fu-PEDOT-NZs primarily arises from indirect ROS modulation and redox stabilization rather than direct cytokine suppression [[76], [77], [78], [79]]. Bioelectricity is a key regulator of inflammation through both neural and cellular pathways. Electrical signaling via the vagus nerve suppresses inflammatory responses by inhibiting major pro-inflammatory cytokines, particularly TNF-α and IL-6, through activation of α7 nicotinic acetylcholine receptors on immune cells. In parallel, direct electrical stimulation can modulate immune cell behavior, promoting a shift from pro-inflammatory (M1) to anti-inflammatory (M2) macrophage phenotypes. Local bioelectric cues also enhance wound healing by improving blood flow, cell migration, angiogenesis, and shortening the inflammatory phase. Together, these mechanisms underpin bioelectronic medicine as a promising, targeted approach for controlling inflammation driven by TNF-α and IL-6 [[80], [81], [82]]. Mechanistically, CHL@Pro-Fu-PEDOT-NZs mitigated DR-associated pathology by downregulating HIF-1α and VEGF expression, consistent with reports that photochemical modulation of hypoxia and redox balance can attenuate neovascularization and oxidative injury.

From a translational perspective, the system's biocompatibility, multifunctionality, and noninvasive administration present a significant advancement over conventional treatments that are often limited by invasiveness or systemic side effects. However, despite these promising results, certain limitations remain. The study was conducted in a preclinical model, and the long-term biosafety, pharmacokinetics, and large-scale reproducibility of the formulation require further validation. Additionally, while the hypothesized pathways for intranasal-to-retinal transport were discussed, direct imaging-based evidence of particle migration should be explored in future work. Collectively, this study positions CHL@Pro-Fu-PEDOT-NZs as a next-generation nanotherapeutic system that bridges photobiology and nanomedicine to achieve precision treatment of ocular diseases.

## 5 Conclusion

In conclusion, we developed an intranasally deliverable, light-activated biohybrid platform (CHL@Pro–Fu–PEDOT-NZs) that integrates photosynthetic microalgae with a conductive PEDOT interface and multifunctional fucoidan-mediated vascular addressing to treat diabetic retinopathy through a multi-pronged mechanism. Under 660 nm irradiation, this system could integrate photosynthesis-driven oxygenation and bioelectron generation with targeted lesion localization, thereby potentially ameliorating hypoxia-associated signaling and vascular pathology while simultaneously suppressing inflammatory and oxidative stress cascades in the diabetic retina.

Importantly, the platform demonstrated favorable safety, which collectively suggest that this strategy can achieve therapeutic action without evident in toxicity.

Looking forward, this work establishes a translatable “nose-to-retina” phototherapeutic paradigm that may reduce reliance on invasive intravitreal injections and enable repeatable, patient-friendly administration. To advance clinical translation, future studies should prioritize (i) long-term and repeated-dose immunogenicity/chronic toxicity profiling (including mucosal immunity and antibody/complement responses), (ii) direct imaging-based validation of intranasal-to-retinal trafficking and pharmacokinetics, and (iii) scalable manufacturing/reproducibility and device-integrated illumination standardization in larger animal models.

## Contributions

The manuscript was written through the contributions of all authors. All authors have approved the final version of the manuscript.

## Ethics approval and consent to participate

This study was approved under guidelines of the Animal Ethics and Use Committee of Taipei Medical University.

## Consent for publication

Every author has reviewed the final manuscript and given full approval for its publication.

## Availability of data and materials

The corresponding author can provide the datasets upon request.

## Declaration of generative AI and AI-assisted technologies in the writing process

During the preparation of this work the authors used ChatGPT to enhance the readability and language of the final paper. Once using this tool, the authors reviewed and edited the content as needed and took full responsibility for the content of the publication.

## CRediT authorship contribution statement

**Andrew E.-Y. Chuang:** Conceptualization, Data curation, Formal analysis, Funding acquisition, Investigation, Methodology, Project administration, Supervision, Validation, Visualization, Writing – original draft, Writing – review & editing. **Yo-Lin Chen:** Conceptualization, Data curation, Formal analysis, Investigation, Methodology, Visualization, Writing – original draft, Writing – review & editing. **Chia-Hung Liu:** Conceptualization, Data curation, Formal analysis, Investigation, Methodology, Writing – original draft, Writing – review & editing. **Hieu Trung Nguyen:** Writing – original draft, Writing – review & editing. **Tsung-Jen Wang:** Investigation. **I-Chan Lin:** Conceptualization, Data curation, Formal analysis, Project administration, Supervision, Validation, Writing – original draft, Writing – review & editing.

## Declaration of competing interest

The authors declare that they have no known competing financial interests or personal relationships that could have appeared to influence the work reported in this paper.

## Data Availability

Data will be made available on request.
