# Supplementary material for: Intranasally delivered fucoidan nanozyme–decorated photosynthetic Chlamydomonas regulates vascular pathology and hypoxia in diabetes-associated retinal microvascular dysfunction
Source: Mater Today Bio. 2026 Mar 26;38:103060. doi: 10.1016/j.mtbio.2026.103060 (PMC13089147; doi:10.1016/j.mtbio.2026.103060)
Supplement: Multimedia component 1 [file mmc1.docx]

**
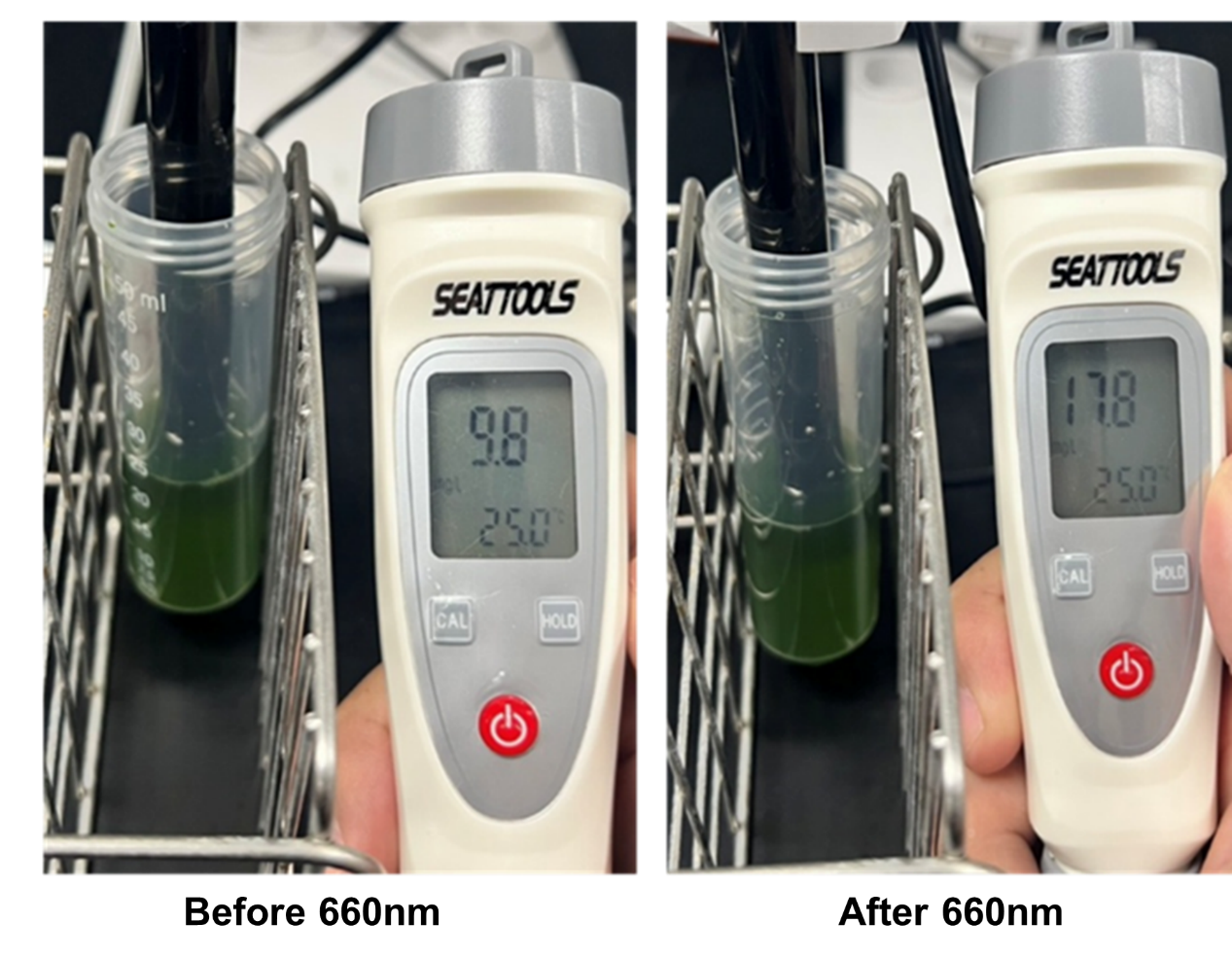
**

**Figure S1.** Dissolved oxygen concentration increased from 9.8 before NIR exposure to 17.8 after irradiation, demonstrating the light-responsive oxygen-producing capacity of CHL.


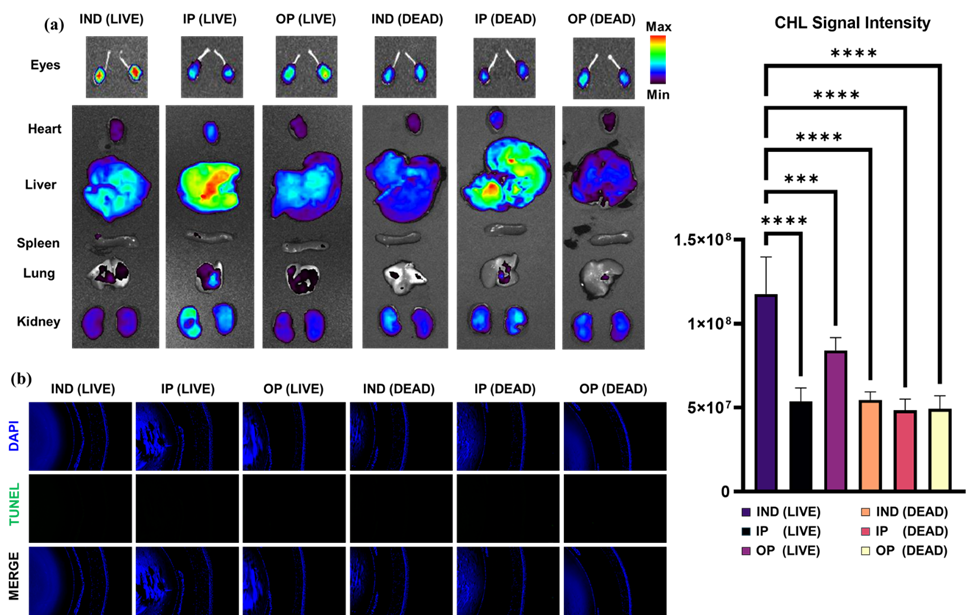


**Figure S2: In vivo and ex vivo fluorescence imaging and apoptotic analysis after different administration routes of CHL.** (a) In vivo and ex vivo fluorescence imaging of eyes and major organs (heart, liver, spleen, lung, kidney) following intranasal delivery (IND, live and dead), intraperitoneal delivery (IP, live and dead), and topical (OP, live and dead). Quantitative analysis of fluorescence intensity indicated significantly higher signals in IND (live) compared to all other groups. Data are presented as mean ± SD. (b) Representative DAPI (blue) and TUNEL (green) staining of eye sections. (c)


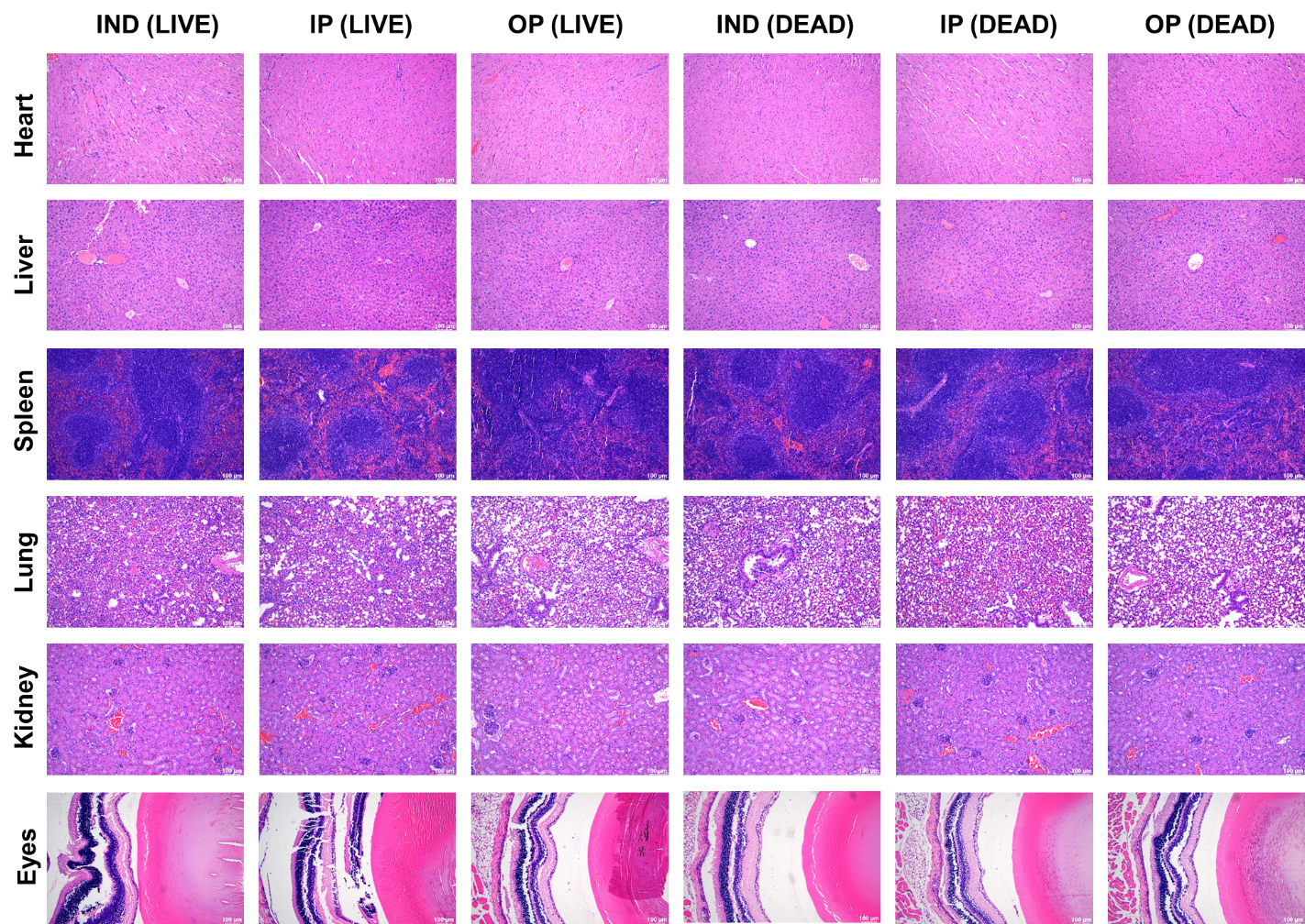


**Figure S3: Histological evaluation of major organs following different delivery methods of CHL.** Representative H&E-stained tissue sections of heart, liver, spleen, lung, kidney, and eye collected from mice treated with live CHL and dead CHL delivery systems. Across all groups, organ morphology remained normal, with no evidence of necrosis, inflammatory infiltration, fibrosis, or structural abnormalities. These results confirm that CHL administration did not induce detectable histopathological changes in major organs.

**
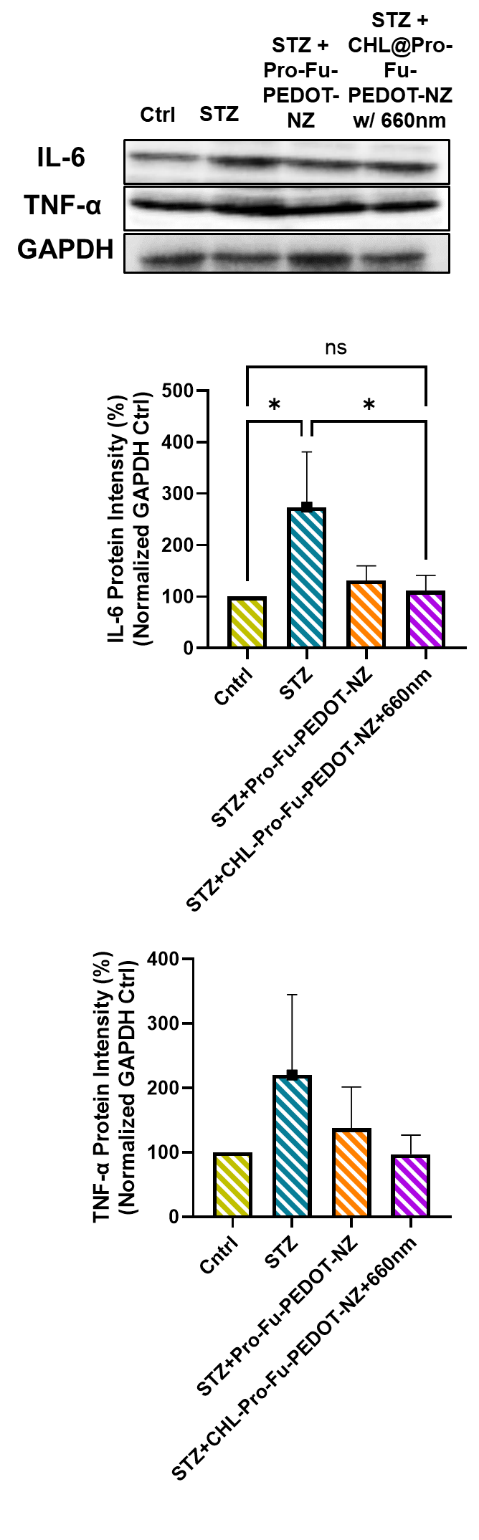
**

**Figure S4. Western blot analysis of IL-6 and TNF-α expression.** Representative blots and densitometric quantification of IL-6 and TNF-α in Control, STZ, STZ + Pro-Fu-PEDOT-NZs, and STZ + CHL@Pro-Fu-PEDOT-NZs w/ NIR groups. Protein levels were normalized to GAPDH and expressed relative to the control (100%). Both IL-6 and TNF-α were markedly upregulated in the STZ group and progressively reduced by treatment, with the greatest suppression observed in the CHL@Pro-Fu-PEDOT-NZs w/ NIR group, approaching baseline levels.
